# Identification and validation a costimulatory molecule gene signature to predict the prognosis and immunotherapy response for hepatocellular carcinoma

**DOI:** 10.1186/s12935-022-02514-0

**Published:** 2022-02-22

**Authors:** Yinan Hu, Jingyi Liu, Jiahao Yu, Fangfang Yang, Miao Zhang, Yansheng Liu, Shuoyi Ma, Xia Zhou, Jingbo Wang, Ying Han

**Affiliations:** 1 Institute of Digestive Diseases, Xijing Hospital, Air Force Medical University, Xi’an, 710032 Shaanxi China; 2 Department of Radiation Oncology, Xijing Hospital, Air Force Medical University, Xi’an, 710032 Shaanxi China

**Keywords:** Hepatocellular carcinoma, Costimulatory molecule, Prognostic signature, ssGSEA, IPS

## Abstract

**Background:**

Hepatocellular carcinoma (HCC) is one of the most common malignancies worldwide. Costimulatory molecules have been proven to be the foundation of immunotherapy. However, the potential roles of costimulatory molecule genes (CMGs) in HCC remain unclear. Our study is aimed to develop a costimulatory molecule-related gene signature that could evaluate the prognosis of HCC patients.

**Methods:**

Based on The Cancer Gene Atlas (TCGA) database, univariate Cox regression analysis was applied in CMGs to identify prognosis-related CMGs. Consensus clustering analysis was performed to stratify HCC patients into different subtypes and compared them in OS. Subsequently, the LASSO Cox regression analysis was performed to construct the CMGs-related prognostic signature and Kaplan–Meier survival curves as well as ROC curve were used to validate the predictive capability. Then we explored the correlations of the risk signature with tumor-infiltrating immune cells, tumor mutation burden (TMB) and response to immunotherapy. The expression levels of prognosis-related CMGs were validated based on qRT-PCR and Human Protein Atlas (HPA) databases.

**Results:**

All HCC patients were classified into two clusters based on 11 CMGs with prognosis values and cluster 2 correlated with a poorer prognosis. Next, a prognostic signature of six CMGs was constructed, which was an independent risk factor for HCC patients. Patients with low-risk score were associated with better prognosis. The correlation analysis showed that the risk signature could predict the infiltration of immune cells and immune status of the immune microenvironment in HCC. The qRT-PCR and immunohistochemical results indicated six CMGs with differential expression in HCC tissues and normal tissues.

**Conclusion:**

In conclusion, our CMGs-related risk signature could be used as a prediction tool in survival assessment and immunotherapy for HCC patients.

**Supplementary Information:**

The online version contains supplementary material available at 10.1186/s12935-022-02514-0.

## Introduction

Primary liver cancer is an aggressive malignant tumor with high mortality worldwide [1]. Hepatocellular carcinoma (HCC) is the most common histological subtype and the fourth leading cause of cancer-related mortality, accounts for approximately 90% of all primary liver cancer. At present, the traditional treatment methods for HCC are systemic chemotherapy, local ablation, TACE (Transhepatic Arterial Chem Otherapy and Embolization) and surgical resection [2]. However, the therapeutic effect of these methods is away from satisfactory. The effect of anti-tumor agents was not consistent in different clinical trials. Portal vein thrombosis and cholestasis or biliary fistula still needed to be addressed in ablation process, when the target lesion was close to vessels and bile ducts [3, 4]. The recurrence rate of liver cancer after resection was up to 70% at 5 years [5]. In recent years, some clinical trials related immunotherapy showed different outcomes in improving the prognosis of HCC patients [6–8]. Therefore, it is urgently required to explore novel prognostic signature for HCC that can predict survival and the response to immunotherapy.

The component of immune microenvironment in HCC is the target for many therapeutic advances, including immunotherapy [9]. Most recently, immunotherapies targeting the adaptive immune system, specifically, T cells, have improved tumor control [10]. Activating T cells involves many signals, among which costimulatory molecules are important [11, 12]. HCC could utilize immune checkpoint and evade anti-tumor immune responses by expressing the corresponding costimulatory ligands [13]. B7-CD28 superfamily is a pivotal signal in co-stimulation of T cell activation, and PD-1/PD-L1 also belong to it, which demonstrated the critical effect of costimulatory molecules in HCC [14, 15]. Besides, accumulating evidence has shown that TNF superfamily, another costimulatory signals, plays a central role in cancer immune regulation [16]. The OX40-OX40L axis, a member of the TNF superfamily, has been shown to improve anti-tumor effects of immune cells and effect for cancer immunotherapy [17–19]. Previous studies also have shown that costimulatory molecules can regulate the tumor immune microenvironment (TME), mainly affecting the activation and proliferation of T cells [20]. Thus, these molecules possibly could provide novel insights in TME. However, the functions of costimulatory molecules in HCC remain unclear.

In this systematic study, we evaluated the expression levels of costimulatory molecules genes in HCC tissues and normal tissues from The Cancer Genome Atlas (TCGA) database. Then a costimulatory molecules-related prognostic signature was constructed for HCC patients and we explored the associations between the prognostic signature and clinicopathological features. Furthermore, we also analyzed the potential roles of this prognostic signature in the immune microenvironment, tumor mutation analysis and response to immunotherapy in different subgroups.

## Materials and methods

### Data collection

The transcriptomic data and corresponding clinical information of HCC were downloaded from the public The Cancer Genome Atlas (TCGA) data portal (https://portal.gdc.cancer.gov/). A total of 50 normal samples and 374 HCC samples were obtained. Patients with incomplete overall survival (OS) information were excluded. Subsequently, the TCGA cohort was randomly divided into training set (n = 186) and test set (n = 184). There were no significantly differences in clinical characteristics between two sets (Table 1). Furthermore, a total of sixty costimulatory molecules genes (CMGs) were collected from prior reviews [21, 22].

**Table 1:** The clinical information in training set, test set and total set

Characteristic	Type	Total set	Test set	Training set	*P* value
Age	≤ 65	232 (62.7%)	116 (63.04%)	116 (62.37%)	0.9782
	> 65	138 (37.3%)	68 (36.96%)	70 (37.63%)	
Gender	Female	121 (32.7%)	61 (33.15%)	60 (32.26%)	0.9422
	Male	249 (67.3%)	123 (66.85%)	126 (67.74%)	
Grade	G1–2	232 (62.7%)	116 (63.04%)	116 (62.37%)	0.9214
	G3–4	133 (35.95%)	65 (35.33%)	68 (36.56%)	
	Unknown	5 (1.35%)	3 (1.63%)	2 (1.08%)	
Stage	Stage I-II	256 (69.19%)	130 (70.65%)	126 (67.74%)	0.2024
	Stage III-IV	90 (24.32%)	38 (20.65%)	52 (27.96%)	
	Unknown	24 (6.49%)	16 (8.7%)	8 (4.3%)	
T	T1–2	274 (74.05%)	140 (76.09%)	134 (72.04%)	0.2946
	T3–4	93 (25.14%)	41 (22.28%)	52 (27.96%)	
	Unknown	3 (0.81%)	3 (1.63%)	0 (0%)	
M	M0	266 (71.89%)	128 (69.57%)	138 (74.19%)	0.573
	M1	4 (1.08%)	3 (1.63%)	1 (0.54%)	
	Unknown	100 (27.03%)	53 (28.8%)	47 (25.27%)	
N	N0	252 (68.11%)	123 (66.85%)	129 (69.35%)	0.1515
	N1–3	4 (1.08%)	0 (0%)	4 (2.15%)	
	Unknown	114 (30.81%)	61 (33.15%)	53 (28.49%)	

### Identification of differentially expressed genes (DEGs)

We utilized “limma” package in R software (version 4.0.4) to identify the differentially expressed genes (DEGs) between all HCC specimens and normal specimens according to the criteria of *P*-value < 0.05 and |log2 (fold change) |> 1. The DEGs were notated with *** if *P* < 0.001, ** if *P* < 0.01 and * if *P* < 0.05. A PPI network was constructed using the Search Tool for the Retrieval of Interacting Genes (STRING) database (http://www.string-db.org/) to explore the interactions between these DEGs.

### Consensus clustering of prognosis-related CMGs

Univariate Cox regression analysis was performed to screen the CMGs with prognostic values in HCC with the cutoff value of *P* < 0.05. To further elucidate the biological characteristics and prognostic values of CMGs, we employed the “ConsensusClusterPlus” package to cluster the HCC patients into different subgroups [23]. Principal Component Analysis (PCA) was performed using R package to assess the distribution of gene expression among different subtypes. The OS difference between different clusters was verified by the Kaplan–Meier curves. Gene set enrichment analysis (GSEA) was conducted in gene set “h.all.v7.2.symbols.gmt” using Java GSEA software (version 4.1.0) to identify the potential biological processes among different clusters. An enrichment pathway with the normalized *P* < 0.05 and the false discovery rate (FDR) value < 0.05 were considered as statistically significant.

### Construction of costimulatory molecule-related prognostic signature

Patients with HCC were randomly divided into a training set and a test set. The training set was used to construct a prognostic costimulatory molecule-related risk signature of HCC, and the test set and total set were used to validate the prognostic power of this risk signature. The least absolute shrinkage and selection operator (LASSO) penalized Cox proportional hazards regression was performed to narrow down the candidate genes and construct the risk model based on the prognosis-related costimulatory molecule genes using the R package “glmnet” [24]. The penalty parameter (λ) was determined by the minimum criteria. The risk score was calculated with the following formular for each patient: Risk score = expression of gene 1 * coefficient 1 + expression of gene 2 * coefficient 2 + expression of gene 3 * coefficient 3 + … + expression of gene n * coefficient n [25]. Patients were divided into high- and low-risk groups according to the median cutoff of the risk score. The area under the curve (AUC) was calculated between high- and low-risk groups with R package “survivalROC” to validate the prognostic capability. The Kaplan–Meier survival curves of the high- and low-risk groups were plotted using R package “survival” and “survminer” to demonstrate the OS of the patients.

### Construction and validation of a nomogram

The nomogram and calibration curves were constructed with R package “rms”. The consistency between the predicted and actual survival of the calibration curves was used to evaluate the accuracy of the nomogram. Meanwhile, the nomogram was examined using the ROC curves.

### Functional enrichment analysis

HCC patients were stratified into high- and low-risk groups based on the median risk score. To explore the potential molecular mechanisms of the risk model genes, DEGs between the high- and low-risk groups were identified with the criteria of |log2FC|≥ 1 and FDR < 0.05. Gene Ontology (GO) and Kyoto Encyclopedia of Genes and Genomes (KEGG) enrichment analyses were conducted using the “clusterProfler” package in R software according to the DEGs.

### Assessment of immune cell infiltration

The Cell-type Identification by Estimating Relative Subsets of RNA Transcripts (CIBERSORT) analysis were performed to estimate the proportions of immune cells infiltration using R package “CIBERSORT” from RNA-sequencing data in TCGA [26]. Wilcoxon rank-sum test was used to examine the differences of infiltrating immune cells in high- and low-risk groups. The tumor microenvironment score was calculated using R package “ESTIMATE” [27].

### Mutation analysis

The mutation data for HCC patients were downloaded from the TCGA data portal (https://portal.gdc.cancer.gov/). Mutation data were further analyzed using the “maftools” package [28]. We calculated the tumor mutation burden (TMB) score for each HCC patient as follows: (total mutation/total covered bases) × 10^6 [29].

### Immunophenoscore analysis

Immunophenoscore (IPS) could well predict the response of immune checkpoint inhibitors (ICIs). The immunogenicity is determined by four major categories of genes, including effector cells, major histocompatibility complex (MHC) molecules, immunomodulators and immunosuppressive cells. The IPS of a patient can be derived using machine learning without bias. The scores of IPS were calculated using a scale ranging from 0–10 based on representative cell type gene expression z-scores. The IPS of every HCC patient was obtained from The Cancer Immunome Atlas (TCIA) (https://tcia.at/home).

### Verification of prognosis-related CMGs expression

Total RNA was extracted from tissue samples using Trizol reagent (Sigma, USA), and then, RNA was reverse transcribed into cDNA with the Evo M-MLV RT Premix (Accurate Biotechnology (Hunan) Co.,Ltd). Quantitative real-time PCR (qRT-PCR) analyses were performed by SYBR Green premix pro Taq HS qRT-PCR kit (Accurate Biotechnology (Hunan) Co.,Ltd) to validate gene expression, and the level of β-Actin served as an internal control. The relative expression was calculated based on the comparative Ct (2^−ΔΔCt^) method. The primers’ sequences for qRT-PCR are shown in Table 2. The protein expression levels of 6 prognostic gene signatures in normal liver and HCC tissues were determined using the Human Protein Atlas (HPA).

**Table 2:** The primer sequences for qRT-PCR analysis

Premier	Sequences (5′–3′)
β-Actin-F	CTCCATCCTGGCCTCGCTGT
β-Actin-R	GCTGTCACCTTCACCGTTCC
TNFSF4-F	CCCTGGGACCTTTGCCTATT
TNFSF4-R	GGGGTTGGACCCTTTCCATC
TNFRSF4-F	AAGCCTGGAGTTGACTGTGC
TNFRSF4-R	CCTGTCCTCACAGATTGCGT
TNFRSF11A-F	GTTGCAGCTCAACAAGGACAC
TNFRSF11A-R	CAGAGAAGAACTGCAAACCGC
TNFRSF11B-F	CTGGAACCCCAGAGCGAAAT
TNFRSF11B-R	GCCTCCTCACACAGGGTAAC
TMIGD2-F	AGAACAGAAACCGGATCGCA
TMIGD2-R	GGCTGTTACCTGAGTCCCTT
CD40LG-F	ATGGGAAACAGCTGACCGTT
CD40LG-R	GATTGTTGCCCGCAAGGTTT

### Tissue collection

Forty-three matched tumorous and non-tumorous tissue specimens of HCC were collected from The Xijing Hospital of Air Force Medical University during 2017–2018. None of the enrolled patients had received any antitumor agents, such as chemotherapeutic agents, targeted agents, or immunotherapy, prior to surgical resection. The clinicopathological details are shown in Table 3. The research was approved by the Institutional Research Ethics Committees of the Xijing Hospital. Informed consent for publication was obtained from all patients for collection of tissue samples prior to the surgery.

**Table 3:** The clinical features of the HCC (n = 43)

Characteristics	Samples (N = 43)	Percentage (%)
Gender		
Male	31	72
Female	12	28
Age		
≤60	35	81
> 60	8	19
Aetiology		
HBV	41	95
Others	2	5
AFP, ng/ml		
< 400	37	86
≥ 400	6	14
T		
T1–2	39	91
T3–4	4	9
N		
N0	42	98
N1	1	2
M		
M0	43	100
M1		
Stage		
Stage I–II	39	91
Stage III–IV	4	9

## Results

### Identification of DEGs between normal and HCC tissues

**Fig. 1:**
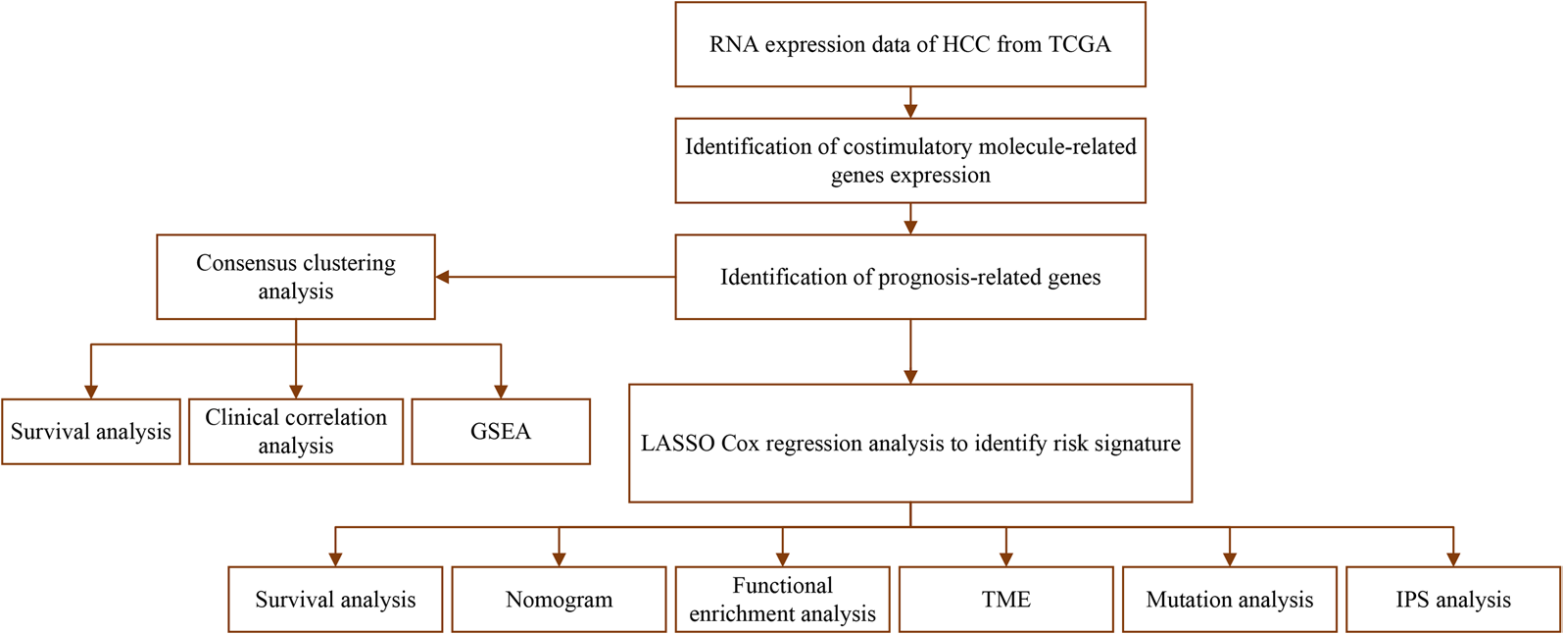
The flowchart of the study

**Fig. 2:**
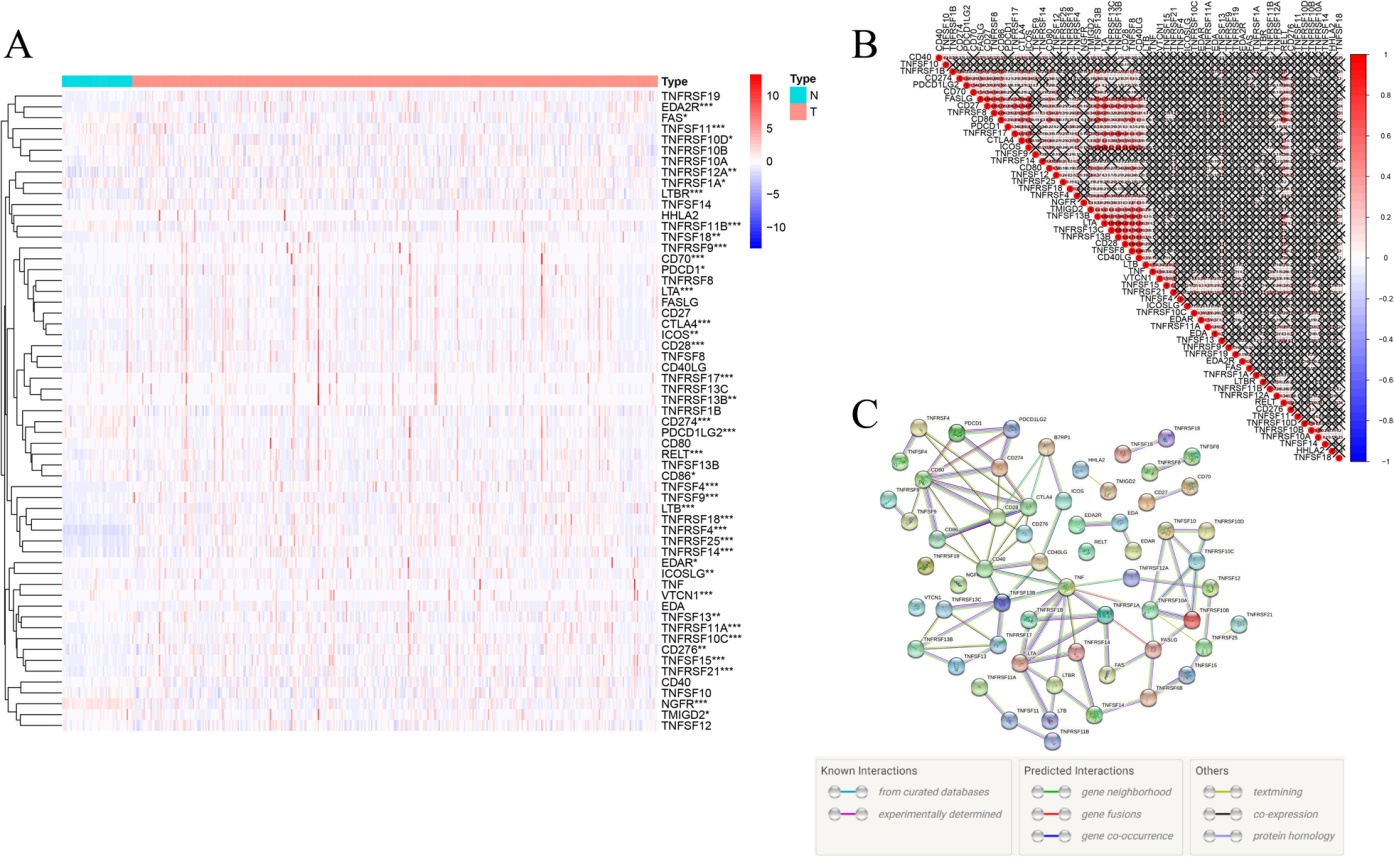
Expressions of the CMGs in HCC. **A** The expression levels of 59 CMGS in both HCC tissues and normal samples. *P < 0.05, **P < 0.01, ***P < 0.001. **B** The correlation of the 59 CMGs by using Spearman correlation analysis. **C** PPI network showed the interactions of the CMGs (the highest confidence: 0.9)

The flowchart of this study was illustrated in Fig. 1. The expression data of 59 CMGs, including 13 well-defined B7-CD28 family costimulatory molecules and 46 TNF family costimulatory molecules genes, were extracted from The Cancer Genome Atlas (TCGA) database after excluding TNFRSF6B for its low expression. The 59 costimulatory molecule-related genes expression levels were compared between HCC tumor and normal tissues, we identified 40 differentially expressed genes (DEGs) (*P* < 0.05). Among these DEGs, 11 genes (NGFR, TNFSF11, PDCD1LG2, CD274, TNFRSF1A, TNFRSF11B, TMIGD2, FAS, TNFRSF10D, TNFSF13 and CD86) were down-regulated while 29 genes (TNFRSF17, TNFRSF13B, CD276, TNFRSF12A, LTBR, TNFSF18, EDAR, TNFRSF14, ICOSLG, RELT, CD28, ICOS, LTA, TNFRSF21, TNFRSF10C, VTCN1, TNFRSF11A, LTB, EDA2RC, TLA4, TNFSF9, TNFRSF25, PDCD1, CD70, TNFSF4, TNFRSF9, TNFRSF18, TNFSF15 and TNFRSF4) were up-regulated in tumor tissues (Fig. 2A and Additional file 1: Table S1). The correlation among CMGs were analyzed. The relationships between each two of them were almost positively correlated, TNFRSF13C and TNFRSF13B were most correlated (Cor = 0.93) (Fig. 2B). A protein–protein interaction (PPI) network was performed to further explore the interactions among these CMGs (Fig. 2C). The minimum required interaction score was set at highest confidence 0.9. The result showed that TNF, CD28, CD40, CD80, CTLA4, LTA, TNFRSF10A and TNFSF13B were hub genes.

### Consensus clustering of prognosis-related CMGs in HCC

**Fig. 3:**
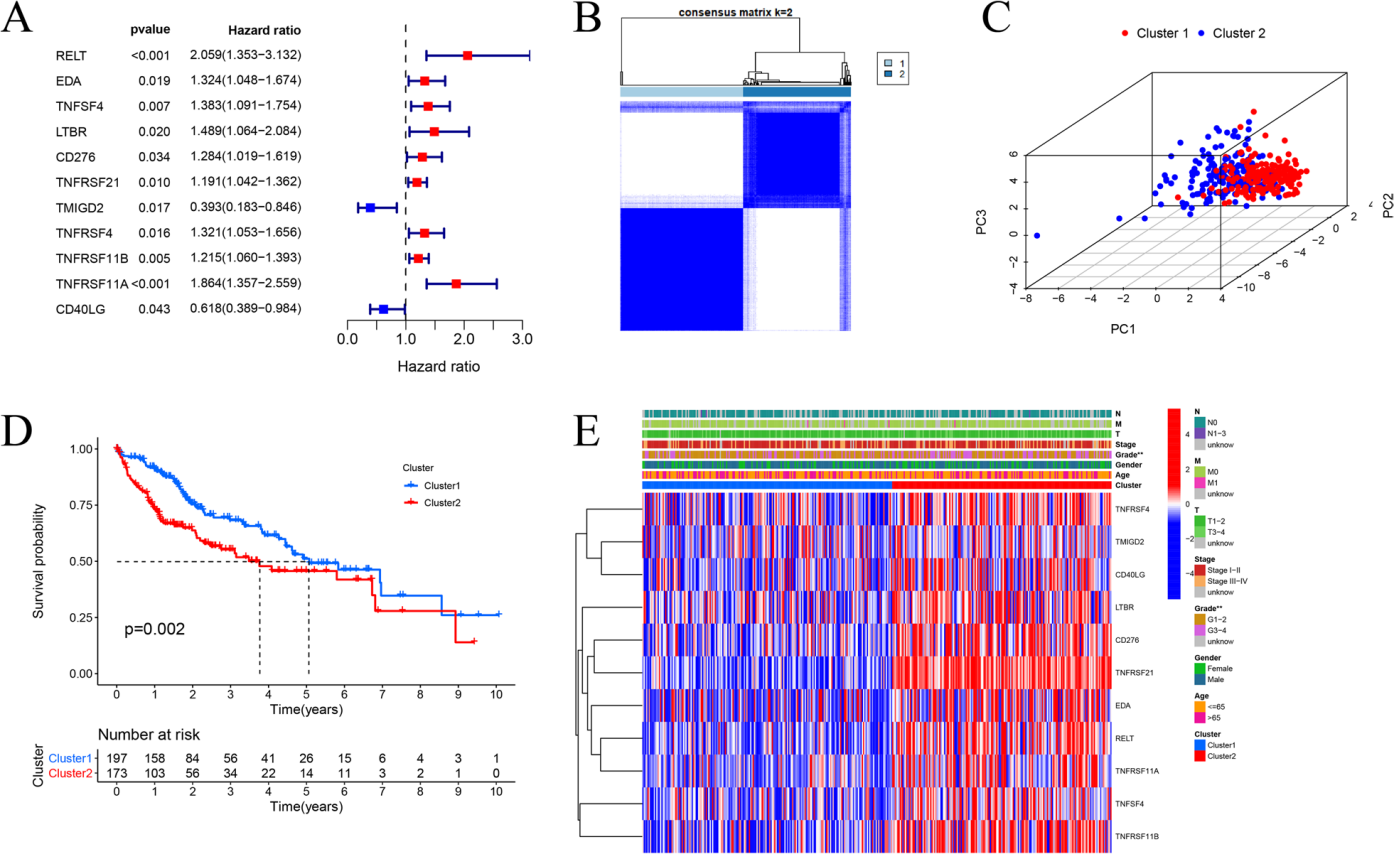
Consensus clustering analysis for HCC patients based on the CMGs. **A **Univariate Cox regression analysis identified prognosis-related CMGs. **B** Consensus clustering matrix for k = 2. **C** Principal Component Analysis (PCA) plot for clusters. **D** Kaplan–Meier overall survival (OS) curves of Cluser1 and Cluster 2. **E** Heatmap and clinical factors of the two clusters

A univariate Cox regression analysis was performed to primary selecting of the survival-related genes from 59 CMGs. A total of 11 CMGs were significantly linked to the prognosis of HCC patients (*P* < 0.05). Two genes were protective genes with hazard ratio (HR) < 1, while 9 genes were risk factors with HR > 1 among them (Fig. 3A). To explore the associations between the expression of 11 prognosis-related CMGs and HCC subtypes, we performed a consensus clustering analysis to classify all HCC patients based on the expression patterns of 11 prognosis-related CMGs. The empirical cumulative density function (CDF) plot is aimed to determine the optimum cluster number (k) from 2 to 9 for the sample distribution to reach an approximate maximum, which means the maximum stability. When k = 2, the consensus matrix showed that HCC patients could be divided into two non-overlapping subgroups with the highest consensus and the clearest cluster partition (Cluster 1: n = 197, Cluster 2: n = 173) (Fig. 3B and Additional file 3: Figure S1A). The PCA were analyzed to verify the reliability between different subgroups, and Cluster 1 and Cluster 2 could gather together and non-overlapped with each other (Fig. 3C). We compared the OS between two subtypes to better understand the relationships between clustering results and survival outcomes, the Kaplan–Meier curves indicated that Cluster 1 had a better prognosis than Cluster 2 (*P* = 0.002, Fig. 3D). The clinical features and two clusters were compared with a heatmap. The majority of 11 prognosis-related CMGs had higher expression in Cluster 2. These two clusters were different in grade (*P* < 0.01), but not with tumor stage, age and gender (Fig. 3E). Furthermore, GSEA analysis showed that oncogenic pathways (apoptosis, G2M-checkpoint, IL2-STAT5-signaling, IL6-JAK-STAT3-signaling, inflammatory response, PI3K-AKT-MTOR-signaling, TNFA-signaling via NF-κB, unfolded protein response) were significantly enriched in Cluster 2 (Additional file 3: Figure S1B).

### Construction and verification of costimulatory molecule-related risk signature

**Fig. 4:**
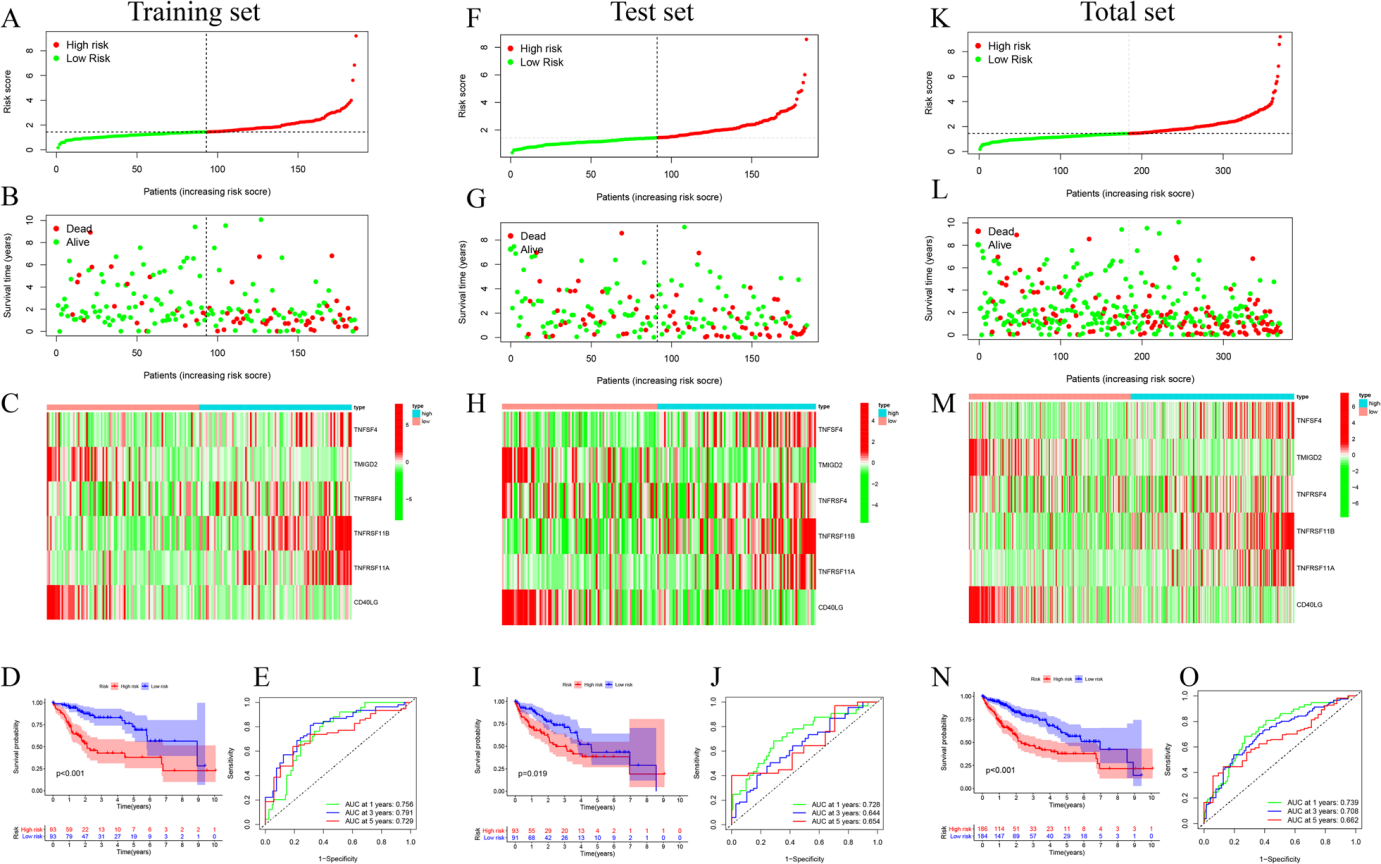
Construction of CMGs risk signature for HCC. **A** The distribution of the risk score, **B** survival status, **C** expression of 6 prognosis-related CMGs in high- and low-risk groups, **D** Kaplan–Meier survival curve, **E** time-dependent ROC curve analyses of the CMGs risk signature in the training set. **F** The distribution of the risk score, **G** survival status, **H** expression of 6 prognosis-related CMGs in high- and low-risk groups, **I** Kaplan–Meier survival curve, **J** time-dependent ROC curve analyses of the CMGs risk signature in the test set. **K** The distribution of the risk score, **L** survival status, **M** expression of 6 prognosis-related CMGs in high- and low-risk groups, **N** Kaplan–Meier survival curve, **O** time-dependent ROC curve analyses of the CMGs risk signature in the total set

To narrow down candidate genes and construct the risk signature, the least absolute shrinkage and selection operator (LASSO) Cox regression analysis was performed in the training set, and 6 of 11 prognosis-related CMGs were identified (Additional file 4: Figure S2). The formula to calculate the risk score as follows: risk score = (0.25584 * TNFSF4) + (−0.29002 * TMIGD2) + (0.13379 * TNFRSF4) + (0.22009 * TNFRSF11B) + (0.40207 * TNFRSF11A) + (−0.78099 * CD40LG). We calculated the risk scores for every HCC patient in the training set according to the above formular. Patients in the training set were divided into high- and low-risk groups based on their median risk sore. A significant difference of OS was observed in different subgroups. High-risk patients had a poorer OS than low-risk groups (*P* < 0.001) (Fig. 4D). Time-dependent receiver operating characteristic (ROC) analysis was used to evaluate the sensitivity and specificity of the risk signature. The areas under the curve (AUC) were 0.756 at 1-year survival, 0.791 at 3-year survival and 0.729 at 5-year survival (Fig. 4E). We ranked the risk scores of patients and analyzed their distribution in the training set (Fig. 4A). The survival status of HCC patients in the training set was showed on the dot plot (Fig. 4B). The heatmap displayed the expressions of 6 prognosis-related CMGs between two risk groups (Fig. 4C).

To determine the stability of the risk signature, we further verified the predictive capability in the test set and total set. The risk score was calculated for each patient in the test set and total set by the same formular obtained from the training set and the patients were classified into high- and low-risk groups. Similarly, Kaplan–Meier survival curve showed significantly difference in two risk groups among the test set. The OS of the high-risk groups was poorer than that of the low-risk groups (*P* = 0.019) (Fig. 4I). The 1-year AUC was 0.728, the 3-year AUC was 0.644 and the 5-year AUC was 0.654 (Fig. 4J). The survival status, the distribution of the risk score and the expression heatmap of 6 prognosis-related CMGs in the test set were presented in Fig. 4F–H.

The results in the total set were similar to the training set and test set. Patients in the high-risk group had a significantly shorter prognosis than patients in the low-risk group (*P* < 0.001) (Fig. 4N). In the total set, the AUC was 0.739 at 1 year, 0.708 at 3 years and 0.662 at 5 years (Fig. 4O). The distribution of the risk score, survival status and the expression patterns of 6 prognosis-related CMGs were showed in Fig. 4K–M.

### Independent prognostic value of the risk signature

**Fig. 5:**
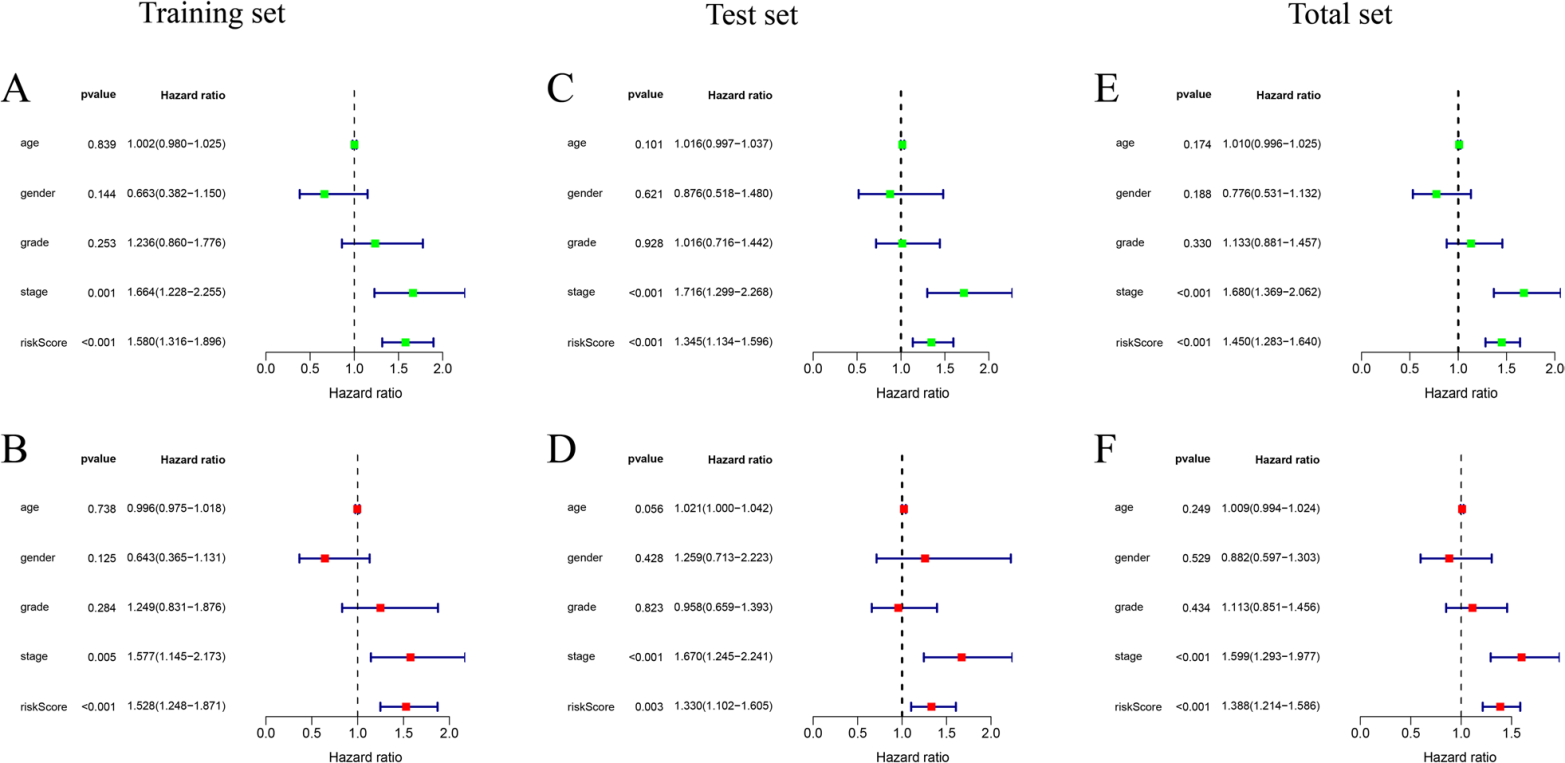
Univariate and multivariate Cox regression analyses. Univariate and Multivariate Cox regression analysis of the correlation between the risk score and clinicopathological features in the training set (**A**, **B**), test set (**C**, **D**) and total set (**E**, **F**), respectively

We performed the univariate and multivariate Cox regression analyses to examine whether the risk score could act as an independent prognosis variable of HCC. Univariate Cox regression analysis showed that pathological tumor stage and risk score were significantly associated with the prognosis (Fig. 5A, C, E). Multivariate Cox regression analyses further identified that the risk score was an independent prognostic factor for OS in the training set, test set and total set (Fig. 5B, D, F).

### Correlations between the risk signature and clinicopathological factors

**Fig. 6:**
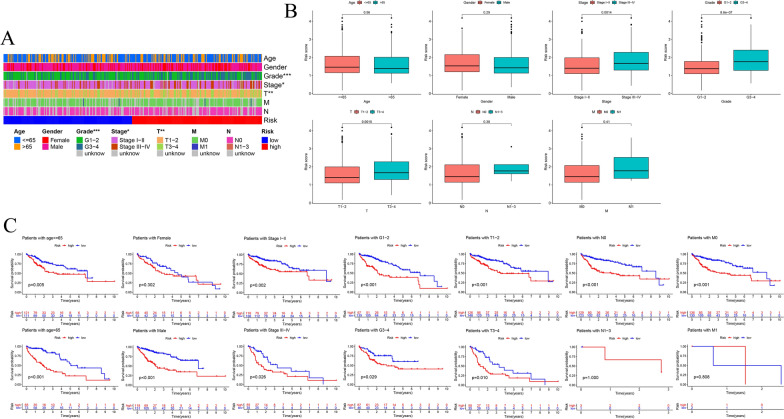
Clinical characteristics of CMGs prognostic signature in different subgroups.** A** The heatmap and clinicopathological factors of high- and low-risk subgroups. *P < 0.05, **P < 0.01, ***P < 0.001. **B** The student’s *t*-test was used to assessed the relationship between the CMGs prognostic signature and age, gender, stage, grade, TMN stage. **C** Kaplan–Meier survival analyses of CMGs risk model in different clinical subgroups based on age, gender, stage, grade, TMN stage with log-rank test

The association between the risk model and clinical characteristics were analyzed to further verify the prognostic value of the risk signature in HCC. The heatmap displayed the expressions of 6 prognosis-related CMGs and the distribution of clinicopathological characteristics in high- and low-risk groups. The risk score was significantly correlated with histological grade, pathological T stage and clinical stage (Fig. 6A). Differences in clinicopathological factors between high- and low-risk groups were showed in Fig. 6B. The risk score of patients with stage III-IV was higher than that of patients with stage I-II (*P* = 0.0014). Patients with G3–4 showed a remarkably higher risk score than those with G1–2 (*P* < 0.001). The risk score in T3–4 patients was significantly higher than that observed in T1–2 patients (*P* = 0.0015). Nonetheless, there were no significant differences among age, gender, N stage and M stage. Furthermore, patients were divided into different subgroups according to the following clinical variables, including age (≤ 65 and > 65), gender (female and male), clinical stage (stage I-II and stage III-IV), grade (G1–2 and G3–4), T stage (T1–2 and T3–4), N stage (N0 and N1–3) and M stage (M0 and M1). The correlation between the risk score and clinicopathological features on OS was explored. Survival analysis manifested that higher risk score were correlated with poor prognosis in age (age ≤ 65 with *P* = 0.005 and age > 65 with *P* < 0.001), gender (*P* < 0.001 in male), stage (stage I-II with *P* = 0.002 and stage III-IV with *P* = 0.026), grade (*P* < 0.001 in G1–2 and *P* = 0.029 in G3–4), T stage (*P* < 0.001 in T1–2 and *P* = 0.01 in T3–4), stage N0 (*P* < 0.001), and stage M0 (*P* < 0.001) (Fig. 6C).

### Construction of a novel nomogram

**Fig. 7:**
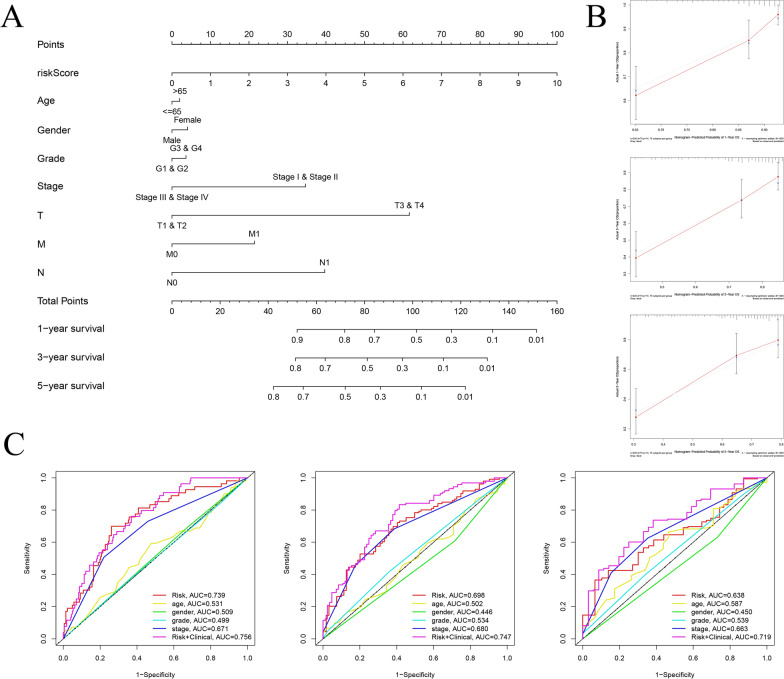
Construction and validation of a novel nomogram. **A** The nomogram for predicting 1-year, 3-year and 5-year OS of HCC in total set. **B** The calibration curves for internal validation of the nomogram on consistency between predicted and observed 1-year, 3-year and 5-year OS in total set. **C** The time-dependent ROC of the nomogram and clinical factors for 1-year, 3-year and 5 year OS prediction in total set

We constructed a nomogram to predict the survival rates for HCC patients based on age, gender, histological grade, TNM stage, tumor stage and risk score (Fig. 7A). The calibration curves of the nomogram indicated good consistency between the predicted survival rate and actual 1-, 3- and 5-year survival rate (Fig. 7B). The AUCs of risk score and tumor stage were 0.739 and 0.671 in 1-year, 0.698 and 0.680 in 3-year, 0.638 and 0.663 in 5-year, respectively (Fig. 7C). These findings suggested that the risk signature might be reliable to predict the OS for HCC patients.

### Functional enrichment analyses based on the risk signature

To explore the potential biological processes for the prognostic risk signature, a total of 474 DEGs were obtained in high- and low-risk groups with the criteria FDR < 0.05 and |log2FC |≥ 1. Among them, 63 genes were downregulated in high-risk group, while 411 genes were upregulated (Additional file 2: Table S2). Gene Ontology (GO) and Kyoto Encyclopedia of Genes and Genomes (KEGG) pathways analyses were carried out based on the DEGs. The results of GO analysis indicated that the DEGs were mainly related to nuclear division. KEGG analysis showed that DEGs were mostly enriched in cell cycle (Additional file 5: Figure S3).

### Association between risk signature and tumor immune microenvironment

**Fig. 8:**
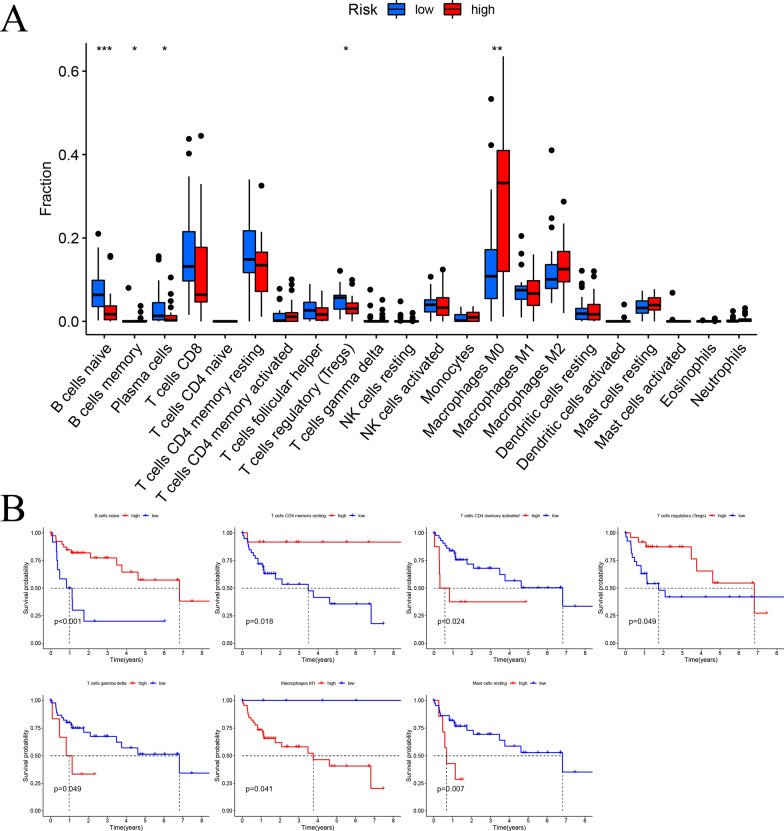
Analysis of tumor infiltrating immune cells. **A** The association of CMGs prognostic signature and immune cells infiltration. Significant statistical differences between two risk groups were assessed by the Wilcoxon rank-sum test, *P < 0.05, **P < 0.01, ***P < 0.001. **B** The relationship between OS and immune cells infiltration (naïve B cells, resting memory CD4 T cells, activated memory CD4 T cells, regulatory T cells, gamma delta T cells, macrophage M1 and resting mast cells)

The differences of tumor-infiltrating immune cells between high- and low-risk groups were analyzed to explore the correlations between the prognostic risk signature and tumor immune microenvironment (TIME). Additional file 6: Figure S4 displayed the abundance of 22 immune cells between high- and low-risk subgroups. Among 22 immune cell types, memory B cells and macrophage M0 were positively correlated with the risk score, while the abundance of naïve B cells, plasma cells and regulatory T cells (Tregs) were significantly enriched in low-risk group (Fig. 8A). Furthermore, the relative proportion of naïve B cells, resting memory CD4 T cells, activated memory CD4 T cells, regulatory T cells, gamma delta T cells, macrophage M1 and resting mast cells were significantly associated with OS (Fig. 8B).

**Fig. 9:**
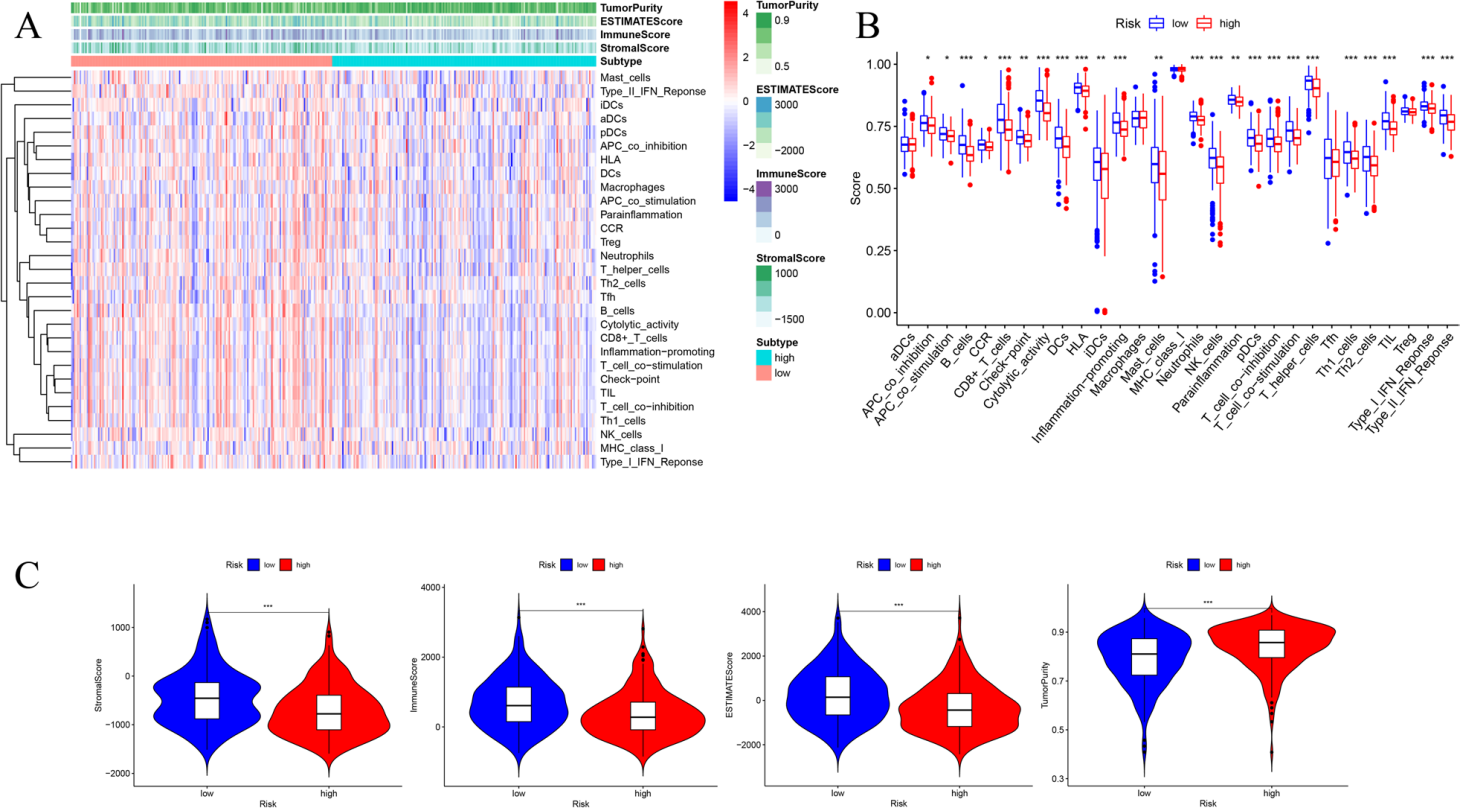
Comparison of the immune status in high- and low-risk groups. **A** The immune status of HCC patients in high- and low-risk groups. Tumor purity, ESTIMATE score, immune score and stromal score of every sample were showed in the heatmap. **B** The box plot displayed the differences of enrichment scores of 16 types of immune cells and 13 immune-related pathways in high- and low-risk groups using Mann–Whitney test. **C** The differences of stromal score, immune score, ESTIMATE score and tumor purity in high- and -risk groups with violin plots. *** *P* < 0.001

To further explore the relationship between the risk signature and immune status, we performed the expression profiles of 29 immune signature gens sets (16 types of immune cells and 13 immune-related pathways) in high- and low-risk groups using the single-sample gene set enrichment analysis (ssGSEA). The heatmap dis1layed the significant differences in immune status between high- and low-risk samples (Fig. 9A). The low-risk subgroup showed higher levels of infiltration of immune cells and higher activity of immune-related pathways (Fig. 9B). We found that the immune score and stromal score were higher in low-risk groups, while the tumor purity was significantly lower in low-risk subgroup (Fig. 9C).

### Differences in molecular characteristics between high- and low-risk groups

**Fig. 10:**
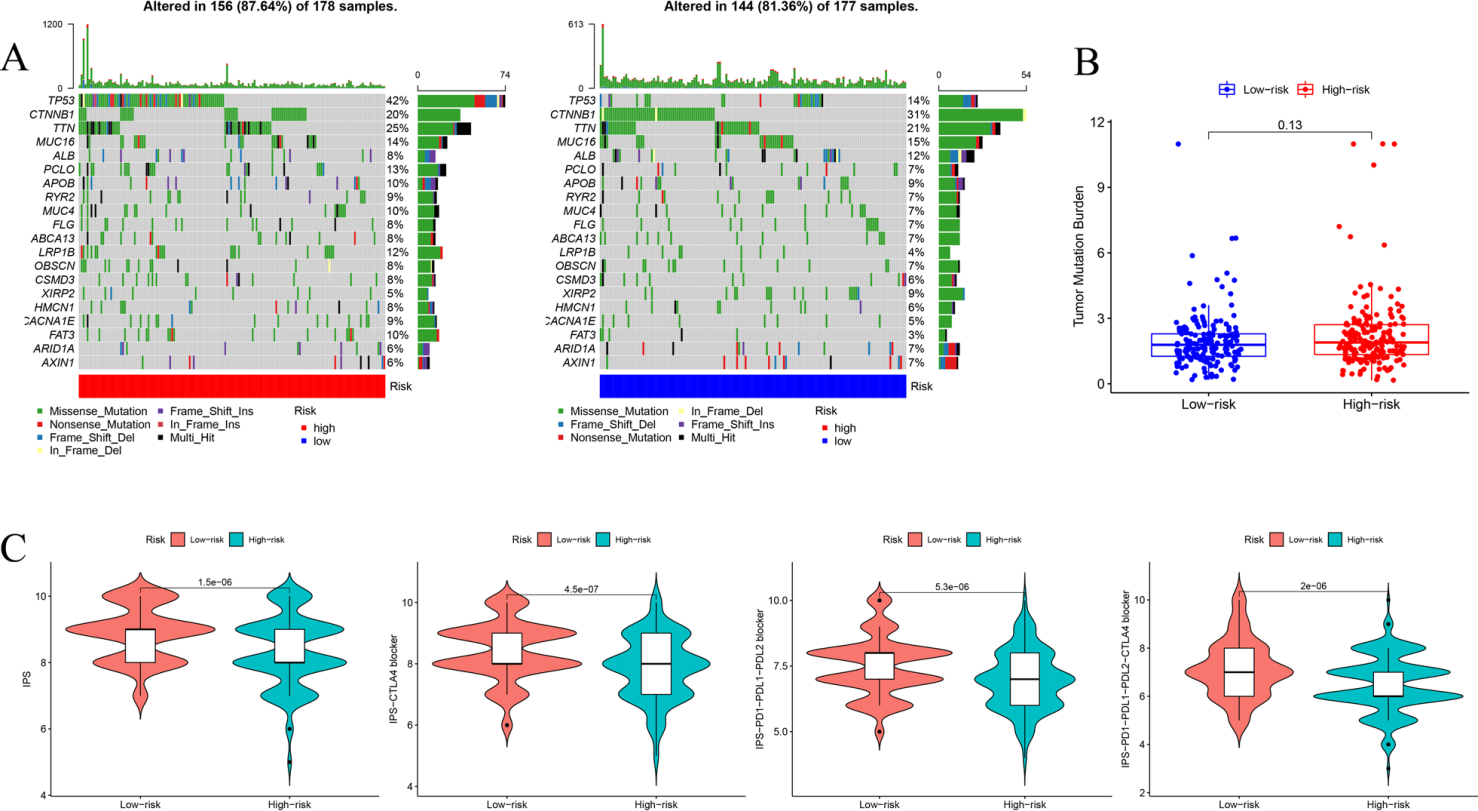
Evaluation of tumor mutation burden and the response to immunotherapy among high- and low-risk groups. **A** Mutation profiles of high- and low-risk groups. **B** The relationship between CMGs risk signature and TMB. **C** The association between IPS and risk signature for HCC patients

We evaluated the relationship between mutation characteristics and the risk signature in TCGA HCC patients with available somatic mutation data. TMB was higher in high-risk patients in spite of no significant difference (Fig. 10B). We also identified the top 20 genes with the highest mutation rates in high- and low-risk subgroups (Fig. 10A). Additionally, we explored the association between immunophenoscore (IPS) and risk signature to predict the potential clinical efficacy and the response to ICI therapy in HCC patient. The IPS, IPS-CTLA4, IPS-PD1-PD-L1- PD-L2, and IPS-PD1-PD-L1-PD-L2-CTLA4 blocker were significantly higher in low-risk group, implying that HCC patients with low-risk score could benefit more from ICI therapy than high-risk patients (Fig. 10C).

### Verification of prognostic CMGs

**Fig. 11:**
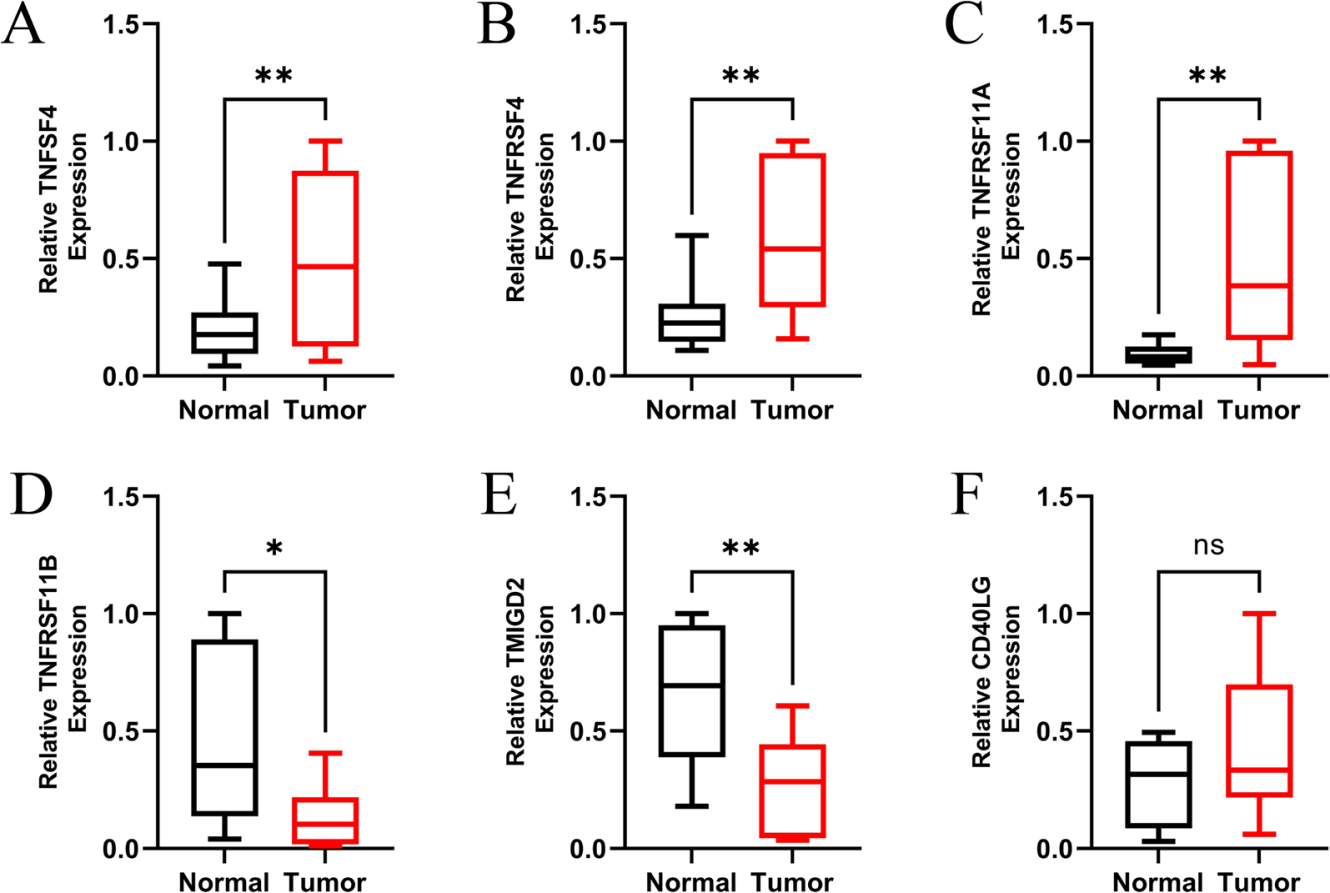
Verification of prognostic genes in HCC tissues and normal tissues. **A** TNFSF4, **B **TNFRSF4, **C** TNFRSF11A, **D** TNFRSF11B, **E** TMIGD2, **F** CD40LG. ns: not significant,*P < 0.05,**P < 0.01

**Fig. 12:**
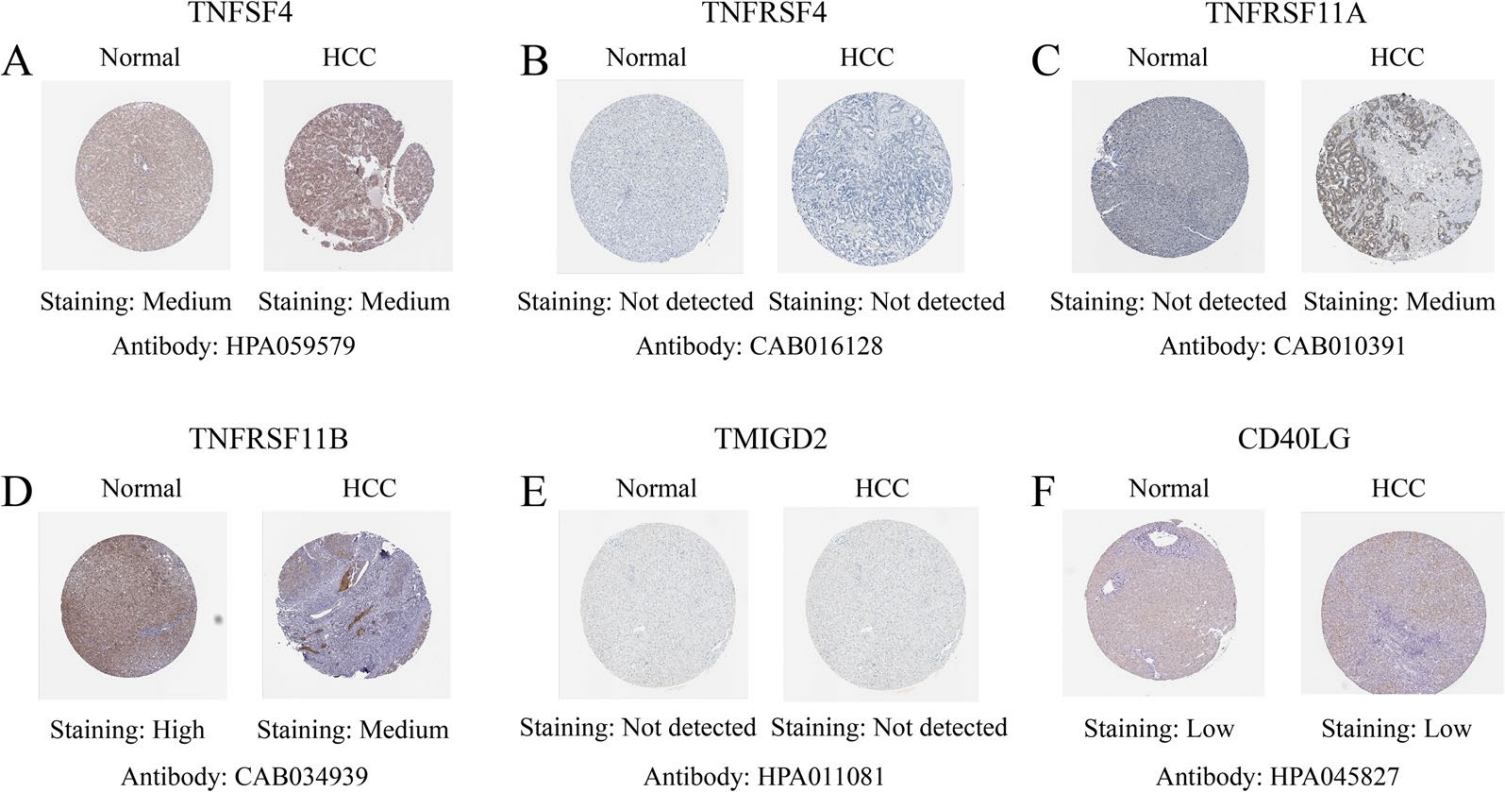
Representative immunohistochemistry images of **A** TNFSF4, **B** TNFRSF4, **C** TNFRSF11A, **D** TNFRSF11B, **E** TMIGD2, **F** CD40LG in HCC and normal liver tissues derived from the HPA database. *HCC* hepatocellular carcinoma, *HPA* Human Protein Atlas

We verified the expression of the CMGs (TNFSF4, TNFRSF4, TMIGD2, TNFRSF11A, TNFRSF11B, CD40LG) in 43 pairs of tumorous and non-tumorous tissue specimens from patients with HCC using qRT-PCR analysis (Fig. 11). The results of qRT-PCR showed that the expression of TNFSF4, TNFRSF4, TNFRSF11A and CD40LG was higher in HCC tissues compared to normal tissues. However, the mRNA expression of TNFRSF11B and TMIGD2 was higher in normal tissues, which was consistent with the results of bioinformatic analysis. The protein expression of the 6 prognostic gene signatures in HCC tissues and normal liver tissues was verified in HPA online database (Fig. 12). The results showed that the expression of TNFRSF11A was increased and the expression of TNFRSF11B was decreased in HCC tissues. TNFRSF4 and TMIGD2 were not detected in hepatocytes. In addition, the staining results of TNFSF4 and CD40LG did not reach significant difference according to HPA database.

## Discussion

Preliminary data from trials of ICIs in the treatment of HCC led to encouraging results. Nevertheless, with the rapid augment in the utilization of ICIs, immune-related adverse events of HCC arose [30, 31]. Multiple studies found that the usage of ICIs strategies targeting costimulatory molecules for HCC management was promising [32]. Therefore, it was necessary to improve the effect on immunotherapy by selecting the suitable HCC patients according to costimulatory molecules expression patterns. In this study, we analyzed the mRNA expression patterns of costimulatory molecules-related in HCC and selected six genes with prognostic values. Then, we constructed the first costimulatory molecule-related prognostic signature for HCC patients. We found that prognostic signature was strongly associated with clinical characteristics. Additionally, our signature was significantly correlated with tumor immune microenvironment and the response to immunotherapy. Univariate and multivariate cox regression analysis indicated our signature could be an independent prognostic factors for the survival of HCC patients. These findings suggested that CMGs risk signature may indicate some insights to personalized targeted treatment in clinical practice.

Costimulatory molecules played an important role in immunotherapy [20]. Recent findings demonstrated that CD28 co-stimulation was necessary for responses to PD-1 blockade in tumor rejection [33]. Thus, understanding the states of costimulatory molecules in HCC patients will help us determine which patients might benefit in immunotherapy. To explore the expression levels of costimulatory molecules in HCC, we acquired 13 members of the B7-CD28 family and 46 members of the TNF family for HCC patients. Six costimulatory molecular genes (TNFSF4, TNFRSF4, TMIGD2, TNFRSF11A, TNFRSF11B, CD40LG) with prognostic values were selected. The TNFRSF4-TNFSF4 pathway provided crucial co-stimulatory signals for CD4^+^T cell responses [34]. Previous study showed that TNFSF4 was closely related to the unfavorable prognosis of HCC patients [35]. In addition, TNFRSF4 was overexpressed in HCC, associated with a more aggressive phenotype and the activation of multiple immunosuppressive pathways [36]. A phase I clinical research also supported that Ivuxolimab (a TNFRSF4 agonist) showed well tolerance and effective anti-tumor capacity in locally advanced or metastatic cancers, including HCC [37]. Consequently, treatment targeting TNFRSF4-TNFSF4 should be considered in the future. TMIGD2 was mainly expressed in tissue-resident lymphocyte T cells, related to improved tumor prognosis [38]. The different interaction between TMIGD2 and B7-H5 have been identified in certain cancers, such as lung cancer, osteosarcoma, oral squamous cell carcinoma (OSCC), colorectal cancer (CRC) and glioma [39]. The TMIGD2-B7-H5 interaction was involving in Akt-dependent signal pathway, which was a recognized regulation in tumor [40]. Previous study reckoned that HCC patients were likely to be benefited from Everolimus (involved in the Akt-MTOR pathway) only after molecular screening [41]. As such, we could make a reasonable speculation that molecular selection was very necessary for the individualized treatment of HCC and our prognostic signature might provide a new method in distinguishing suitable patients. Of note, our results firstly revealed that TMIGD2 was highly expressed in HCC with favorable prognosis. TNFRSF11A, also known as RANK, was significantly up-regulated in HCC, and can lead directly to migration and invasion by its ligand [42]. Interestingly, genetic deletion of TNFRSF11A in thymic epithelial cells resulted in impaired thymic involution and blunted expansion of natural regulatory T (Treg) cells [43]. Additionally, study showed that HCC patients with high serum TNFRSF11B, also known as osteoprotegerin (OPG), level had poorer survival rates compared with HCC patients with low OPG level [44]. CD40 ligand-expressing dendritic cells could induce regression of HCC [45]. In addition, some clinical trials targeting the agonist or antagonist of CD40/CD40LG has showed promising results in different malignancies. Study showed that adenoviral vectors expressing CD40 ligand (AdCD40L) were safe in vivo and could reduce the number of tumor cell infiltration in bladder cancer [46]. Besides, AdCD40L intratumoral injection increased T-effector/T-regulatory cell ratio by improving systemic immune condition, which was related favorable survival in malignant melanoma patients [47]. Thus, combined other scholar studies and our own bioanalysis, it was possible to improve the prognosis of HCC patients with a similar approach. Moreover, the expression levels of six prognostic genes were verified using qRT-PCR and immunohistochemistry. However, the protein expression of prognostic genes signatures was not completely similar with our previous results, may partly owing to different race and clinical characteristics. At the same time, it also explained that why targeted therapy and immunotherapy did not work for all HCC patients and tumor heterogeneity should be considered in treatment practice. With these six costimulatory molecular genes elucidated in immunity, we hope that the signature constructed by these could predict the response to immunotherapy for HCC patients.

Tumors were complex ecosystems, defined by spatiotemporal interactions among heterogeneous cell types [48]. Subsequently, we compared the associations between our signature and tumor immune microenvironment, the immune cell infiltration and tumor mutation profiles in high-risk and low-risk patients. Our results showed that naïve B cells, plasma cells and regulatory T cells (Tregs) were significantly enriched in low-risk groups. Much of researches regarded Tregs as an immunosuppressive cell, posing anti-tumor immunity in various cancers [49]. Nevertheless, some scholars stated that inhibiting the expression of PD-1 promoted other immune checkpoints, resulting in impaired immune killing ability [50, 51]. Accordingly, fewer Tregs cells in HCC patients with poor prognosis, indicated that those were more likely to be inhibited by PD-1 and activated more immune checkpoints. Correspondingly, high-risk subgroup manifested lower levels of infiltration of immune cells, implicating less process in immune activation.

Tumor mutation burden (TMB) is emerging as a potential biomarker, and participated in immunotherapy-related pathway [52, 53]. We found that the tumor mutation burden (TMB) in the high-risk group was higher than that in the low-risk group with no significant, partly due to the small sample size. TP53 mutation frequency was evidently higher in high-risk group (frequency rate 42%) than low-risk group (frequency rate 14%) according to our mutation results, suggesting more increases genomic instability and complicated major pathway signaling changes in HCC. Additionally, it was important to highlight that different microenvironment-based immune subtypes, based on gene profiling or signatures, and other molecular features, may help identify subgroups of patients more likely to benefit from specific therapies [54]. Some scholars have found that immune-excluded tumors in HCC were proposed to be primarily resistant to ICIs [55]. IPS could predict the response to immunotherapy in cervical cancer and HCC. The prediction of IPS has been demonstrated in different studies [56, 57]. In the present study, we found low-risk group tended to have higher IPS-CTLA4, IPS-PD1/PD-L1/ PD-L2, and IPS-PD1/PD-L1/PD-L2^+^CTLA4, implying that HCC patients with low-risk score could benefit more from immunotherapy than high-risk patients. Therefore, our signature was of great help to clinical immunotherapy decision.

However, there were some limitations in this study. Firstly, we did not explore the exact function of six costimulatory molecule genes in HCC. Thus, it was still necessary to clarify the mechanism of them in the future. Secondly, it was inevitable that there were limited clinical information for HCC patients in public datasets, so the values of the prognostic signature needed to be determined by experimental and prospective studies. Moreover, the risk signature for evaluating the response to immunotherapy was restricted to costimulatory molecule genes and tumor immune microenvironment was highly heterogeneous. Therefore, the prognostic information for HCC patients with immunotherapy were needed to validate the prediction power of our signature clinically.

## Conclusion

In our study, we first elucidated the expression of costimulatory molecules for HCC patients, and constructed a six CMGs prognostic signature. The costimulatory molecular-related signature could stratify patients into different subsets with adverse clinical outcomes. In addition, immunotherapy response prediction by our signature explained disparate effect on HCC patients. Consequently, we believed our research manifested the capacity of costimulatory molecules and provided clinicians with applicable treatment.

## Supplementary Information

**Additional file 1:** **Table S1**: The differentially expressed costimulatory molecule-related genes in TCGA database.

**Additional file 2:** **Table S2**: The DEGs between high- and low-risk groups in TCGA cohort.

**Additional file 3: Figure S1**: Consensus clustering based on the CMGs. (A) Cumulative distribution function (CDF) curves in consensus clustering and relative changes in area under CDF curves for k = 2 to 10. (B) The Gene Set Enrichment Analysis of the oncogenic pathways in Cluster 2.

**Additional file 4: Figure S2**: A LASSO Cox regression analysis further fine-turned the selection of CMGs-related risk signature.

**Additional file 5: Figure S3**: Functional enrichment analysis of the DEGs. (A) Gene ontology analysis. (B) Kyoto Encyclopedia of Genes and Genomes pathways.

**Additional file 6: Figure S4**: The abundance of 22 immune cells between the high- and low-risk groups.

## Data Availability

Gene expression profiles, clinical information and mutation data of LIHC in this study are available from the public database (TCGA, https:// portal. gdc. cancer. gov/).

## References

[1] Sung H, Ferlay J, Siegel RL, Global cancer statistics, (2020). GLOBOCAN estimates of incidence and mortality worldwide for 36 cancers in 185 countries. CA Cancer J Clin.

[2] Llovet JM, Zucman-Rossi J, Pikarsky E (2016). Hepatocellular carcinoma. Nat Rev Dis Primers.

[3] Ding J, Jing X, Liu J (2013). Complications of thermal ablation of hepatic tumours: comparison of radiofrequency and microwave ablative techniques. Clin Radiol.

[4] Ringe KI, Lutat C, Rieder C (2015). Experimental evaluation of the heat sink effect in hepatic microwave ablation. PLoS ONE.

[5] Franssen B, Alshebeeb K, Tabrizian P (2014). Differences in surgical outcomes between hepatitis B- and hepatitis C-related hepatocellular carcinoma: a retrospective analysis of a single North American center. Ann Surg.

[6] El-Khoueiry AB, Sangro B, Yau T (2017). Nivolumab in patients with advanced hepatocellular carcinoma (CheckMate 040): an open-label, non-comparative, phase 1/2 dose escalation and expansion trial. Lancet.

[7] Zhu AX, Finn RS, Edeline J (2018). Pembrolizumab in patients with advanced hepatocellular carcinoma previously treated with sorafenib (KEYNOTE-224): a non-randomised, open-label phase 2 trial. Lancet Oncol.

[8] Lee MS, Ryoo B-Y, Hsu C-H (2020). Atezolizumab with or without bevacizumab in unresectable hepatocellular carcinoma (GO30140): an open-label, multicentre, phase 1b study. Lancet Oncol.

[9] Sperandio RC, Pestana RC, Miyamura BV (2021). Hepatocellular carcinoma immunotherapy. Annu Rev Med.

[10] Egen JG, Ouyang W, Wu LC (2020). Human anti-tumor immunity: insights from immunotherapy clinical trials. Immunity.

[11] McHayleh W, Bedi P, Sehgal R (2019). Chimeric antigen receptor t-cells: the future is now. J Clin Med.

[12] Chapman NM, Boothby MR, Chi H (2020). Metabolic coordination of T cell quiescence and activation. Nat Rev Immunol.

[13] Chen L, Flies DB (2013). Molecular mechanisms of T cell co-stimulation and co-inhibition. Nat Rev Immunol.

[14] Esensten JH, Helou YA, Chopra G (2016). CD28 costimulation: from mechanism to therapy. Immunity.

[15] Sangro B, Sarobe P, Hervas-Stubbs S (2021). Advances in immunotherapy for hepatocellular carcinoma. Nat Rev Gastroenterol Hepatol.

[16] Croft M (2009). The role of TNF superfamily members in T-cell function and diseases. Nat Rev Immunol.

[17] Lu Y, Zhang M, Wang S (2014). p38 MAPK-inhibited dendritic cells induce superior antitumour immune responses and overcome regulatory T-cell-mediated immunosuppression. Nat Commun.

[18] Aberg KM, Radek KA, Choi EH (2007). Psychological stress downregulates epidermal antimicrobial peptide expression and increases severity of cutaneous infections in mice. J Clin Invest.

[19] Shibahara I, Saito R, Zhang R (2015). OX40 ligand expressed in glioblastoma modulates adaptive immunity depending on the microenvironment: a clue for successful immunotherapy. Mol Cancer.

[20] Wei SC, Duffy CR, Allison JP (2018). Fundamental mechanisms of immune checkpoint blockade therapy. Cancer Discov.

[21] Aye L, Song X, Yang J (2021). Identification of a costimulatory molecule gene signature to predict survival and immunotherapy response in head and neck squamous cell carcinoma. Front Cell Dev Biol.

[22] Hua X, Ge S, Zhang J (2021). A costimulatory molecule-related signature in regard to evaluation of prognosis and immune features for clear cell renal cell carcinoma. Cell Death Discov.

[23] Wang W, Sun B, Xia Y (2020). RNA N6-methyladenosine-related gene contribute to clinical prognostic impact on patients with liver cancer. Front Genet.

[24] Wang H, Lengerich BJ, Aragam B (2019). Precision Lasso: accounting for correlations and linear dependencies in high-dimensional genomic data. Bioinformatics.

[25] Hsuan-Yu C, Sung-Liang Y, Chun-Houh C (2007). A five-gene signature and clinical outcome in non–small-cell lung cancer. N Engl J Med..

[26] Becht E, Giraldo NA, Lacroix L (2016). Estimating the population abundance of tissue-infiltrating immune and stromal cell populations using gene expression. Genome Biol.

[27] Yoshihara K, Shahmoradgoli M, Martinez E (2013). Inferring tumour purity and stromal and immune cell admixture from expression data. Nat Commun.

[28] Mayakonda A, Lin DC, Assenov Y (2018). Maftools: efficient and comprehensive analysis of somatic variants in cancer. Genome Res.

[29] Robinson DR, Wu YM, Lonigro RJ (2017). Integrative clinical genomics of metastatic cancer. Nature.

[30] Finn RS, Ryoo BY, Merle P, Bouattour M, Lim HY, Breder V, Edeline J, Chao Y, Ogasawara S, Yau T, Garrido M (2020). Pembrolizumab as second-line therapy in patients with advanced hepatocellular carcinoma in KEYNOTE-240: a randomized, double-blind, phase III trial. J Clin Oncol..

[31] Lyon AR, Yousaf N, Battisti NML (2018). Immune checkpoint inhibitors and cardiovascular toxicity. Lancet Oncol.

[32] Chinnadurai R, Scandolara R, Alese OB (2020). Correlation patterns among B7 family ligands and tryptophan degrading enzymes in hepatocellular carcinoma. Front Oncol.

[33] Kamphorst AO, Wieland A, Nasti T, Yang S, Zhang R, Barber DL, Konieczny BT, Daugherty CZ, Koenig L, Yu K, Sica GL (2017). Rescue of exhausted CD8 T cells by PD-1–targeted therapies is CD28-dependent. Science..

[34] Gajdasik DW, Gaspal F, Halford EE (2020). Th1 responses in vivo require cell-specific provision of OX40L dictated by environmental cues. Nat Commun.

[35] Hong WF, Gu YJ, Wang N (2021). Integrative characterization of immune-relevant genes in hepatocellular carcinoma. J Clin Transl Hepatol.

[36] Xie K, Xu L, Wu H (2018). OX40 expression in hepatocellular carcinoma is associated with a distinct immune microenvironment, specific mutation signature, and poor prognosis. Oncoimmunology.

[37] Diab A, Hamid O, Thompson JA (2021). A Phase I, open-label, dose-escalation study of the OX40 agonist ivuxolimab in patients with locally advanced or metastatic cancers. Clin Cancer Res.

[38] Tian Y, Sun Y, Gao F (2019). CD28H expression identifies resident memory CD8 + T cells with less cytotoxicity in human peripheral tissues and cancers. Oncoimmunology.

[39] Zhong C, Lang Q, Yu J (2020). Phenotypical and potential functional characteristics of different immune cells expressing CD28H/B7-H5 and their relationship with cancer prognosis. Clin Exp Immunol.

[40] Zhu Y, Yao S, Iliopoulou BP (2013). B7–H5 costimulates human T cells via CD28H. Nat Commun.

[41] Koeberle D, Dufour JF, Demeter G (2016). Sorafenib with or without everolimus in patients with advanced hepatocellular carcinoma (HCC): a randomized multicenter, multinational phase II trial (SAKK 77/08 and SASL 29). Ann Oncol.

[42] Song FN, Duan M, Liu LZ (2014). RANKL promotes migration and invasion of hepatocellular carcinoma cells via NF-kappaB-mediated epithelial-mesenchymal transition. PLoS ONE.

[43] Paolino M, Koglgruber R, Cronin SJF (2021). RANK links thymic regulatory T cells to fetal loss and gestational diabetes in pregnancy. Nature.

[44] Zhang C, Lin J, Ni X (2021). Prognostic value of serum osteoprotegerin level in patients with hepatocellular carcinoma following surgical resection. Front Oncol.

[45] Gonzalez-Carmona MA, Lukacs-Kornek V, Timmerman A (2008). CD40ligand-expressing dendritic cells induce regression of hepatocellular carcinoma by activating innate and acquired immunity in vivo. Hepatology.

[46] Malmstrom PU, Loskog AS, Lindqvist CA (2010). AdCD40L immunogene therapy for bladder carcinoma–the first phase I/IIa trial. Clin Cancer Res.

[47] Schiza A, Wenthe J, Mangsbo S (2017). Adenovirus-mediated CD40L gene transfer increases Teffector/Tregulatory cell ratio and upregulates death receptors in metastatic melanoma patients. J Transl Med.

[48] Sun Y, Wu L, Zhong Y (2021). Single-cell landscape of the ecosystem in early-relapse hepatocellular carcinoma. Cell.

[49] Zheng C, Zheng L, Yoo JK (2017). Landscape of infiltrating T cells in liver cancer revealed by single-cell sequencing. Cell.

[50] Ellestad KK, Thangavelu G, Ewen CL (2014). PD-1 is not required for natural or peripherally induced regulatory T cells: Severe autoimmunity despite normal production of regulatory T cells. Eur J Immunol.

[51] Huang RY, Francois A, McGray AR (2017). Compensatory upregulation of PD-1, LAG-3, and CTLA-4 limits the efficacy of single-agent checkpoint blockade in metastatic ovarian cancer. Oncoimmunology.

[52] Huang T, Yan T, Chen G (2021). Development and validation of a gene mutation-associated nomogram for hepatocellular carcinoma patients from four countries. Front Genet.

[53] Donehower LA, Soussi T, Korkut A (2019). Integrated analysis of TP53 gene and pathway alterations in the cancer genome atlas. Cell Rep.

[54] Sia D, Jiao Y, Martinez-Quetglas I, Kuchuk O, Villacorta-Martin C, de Moura MC, Putra J, Camprecios G, Bassaganyas L, Akers N, Losic B (2017). Identification of an immune-specific class of hepatocellular carcinoma, based on molecular features. Gastroenterology..

[55] Galarreta M, Bresnahan E, Molina-Sanchez P (2019). Beta-catenin activation promotes immune escape and resistance to anti-PD-1 therapy in hepatocellular carcinoma. Cancer Discov..

[56] Yang S, Wu Y, Deng Y (2019). Identification of a prognostic immune signature for cervical cancer to predict survival and response to immune checkpoint inhibitors. Oncoimmunology.

[57] Xu Y, Wang Z, Li F (2021). Survival prediction and response to immune checkpoint inhibitors: a prognostic immune signature for hepatocellular carcinoma. Transl Oncol.

