# Supplementary material for: Identification and validation a costimulatory molecule gene signature to predict the prognosis and immunotherapy response for hepatocellular carcinoma
Source: Cancer Cell Int. 2022 Feb 22;22:97. doi: 10.1186/s12935-022-02514-0 (PMC8864933; doi:10.1186/s12935-022-02514-0)
Supplement: Supplementary file 1 — Additional file 1: Table S1: The differentially expressed costimulatory molecule-related genes in TCGA database. [file 12935_2022_2514_MOESM1_ESM.docx]

| Gene | conMean | treatMean | logFC | pValue |
| --- | --- | --- | --- | --- |
| TNFRSF4 | 0.297797471 | 2.188369572 | 2.877452992 | 6.79E-26 |
| TNFRSF25 | 0.385561902 | 1.71088477 | 2.149708187 | 3.26E-22 |
| TNFSF4 | 0.190816467 | 1.02407932 | 2.424069882 | 1.01E-18 |
| NGFR | 6.55944478 | 2.597936305 | -1.336207644 | 8.26E-18 |
| VTCN1 | 0.939891276 | 2.687972575 | 1.515952634 | 6.39E-14 |
| TNFRSF18 | 0.229587933 | 1.355523556 | 2.561731465 | 9.11E-14 |
| TNFRSF14 | 5.39200134 | 9.313237658 | 0.788461939 | 6.25E-11 |
| LTBR | 14.10823734 | 20.86563194 | 0.564590864 | 3.29E-10 |
| TNFSF15 | 0.05689787 | 0.349877341 | 2.620402681 | 6.61E-09 |
| PDCD1LG2 | 0.803003466 | 0.626770503 | -0.357468928 | 2.38E-08 |
| TNFRSF11B | 3.680970612 | 3.219964546 | -0.193041428 | 2.90E-08 |
| TNFSF11 | 0.6540519 | 0.455283724 | -0.522639235 | 2.24E-07 |
| TNFRSF17 | 0.285446568 | 0.329842353 | 0.208555944 | 5.48E-07 |
| CTLA4 | 0.179602838 | 0.589592527 | 1.714908093 | 6.29E-06 |
| CD28 | 0.236213634 | 0.436234423 | 0.885011377 | 6.67E-06 |
| CD70 | 0.060036918 | 0.321406834 | 2.420478791 | 1.61E-05 |
| LTA | 0.109635358 | 0.265390082 | 1.275401304 | 1.78E-05 |
| CD274 | 0.662843808 | 0.530409432 | -0.321562525 | 5.16E-05 |
| RELT | 0.287985378 | 0.522325757 | 0.858954285 | 7.20E-05 |
| TNFRSF21 | 3.951793894 | 9.953828093 | 1.332743766 | 0.000119117 |
| TNFRSF10C | 0.338443064 | 0.858012755 | 1.342085946 | 0.000121529 |
| EDA2R | 0.15074555 | 0.492836245 | 1.708992946 | 0.000210548 |
| TNFRSF9 | 0.083156895 | 0.490483311 | 2.560296257 | 0.000210548 |
| LTB | 2.754408002 | 8.559166914 | 1.635728105 | 0.000296713 |
| TNFRSF11A | 0.145428148 | 0.418906513 | 1.526321785 | 0.000296713 |
| TNFSF9 | 0.133011038 | 0.549738665 | 2.047199976 | 0.000331567 |
| TNFSF13 | 1.322888628 | 1.297673731 | -0.02776391 | 0.001366689 |
| CD276 | 7.84077528 | 10.96671697 | 0.484063483 | 0.001592052 |
| TNFRSF12A | 21.62263072 | 30.85555553 | 0.51298821 | 0.001731572 |
| TNFRSF13B | 0.055472379 | 0.065603224 | 0.241997109 | 0.002750447 |
| TNFSF18 | 0.090945597 | 0.14809585 | 0.70345552 | 0.003517889 |
| ICOSLG | 0.078610361 | 0.139435424 | 0.826805751 | 0.004403557 |
| ICOS | 0.148099157 | 0.283853097 | 0.938581052 | 0.004522746 |
| TNFRSF10D | 3.479260804 | 3.37611093 | -0.043418519 | 0.010837897 |
| EDAR | 0.19287621 | 0.331032265 | 0.779296629 | 0.016820433 |
| PDCD1 | 0.292666456 | 1.29929548 | 2.150400253 | 0.016990412 |
| TMIGD2 | 0.254465857 | 0.228820605 | -0.153255126 | 0.032506883 |
| TNFRSF1A | 38.2198152 | 33.3451307 | -0.196844699 | 0.034609914 |
| FAS | 3.60335702 | 3.464695826 | -0.056612901 | 0.041556198 |
| CD86 | 1.481166817 | 1.470144694 | -0.010775979 | 0.046050522 |
| TNF | 0.249332429 | 0.245633103 | -0.021565534 | 0.052418904 |
| TNFRSF1B | 13.95443388 | 13.00536722 | -0.101616461 | 0.056910189 |
| FASLG | 0.262519062 | 0.336747002 | 0.359242918 | 0.069820808 |
| TNFRSF8 | 0.070366534 | 0.120856619 | 0.78033513 | 0.072222833 |
| TNFRSF10A | 1.52446141 | 1.962770195 | 0.364591639 | 0.078785847 |
| TNFRSF19 | 0.326601884 | 2.470933053 | 2.919450904 | 0.129283557 |
| CD40LG | 0.404102088 | 0.463194108 | 0.196897097 | 0.183263713 |
| CD40 | 5.60159726 | 7.52209995 | 0.425297215 | 0.379295793 |
| TNFRSF13C | 0.135428309 | 0.217827452 | 0.685656439 | 0.399602486 |
| TNFSF8 | 0.304746859 | 0.310582796 | 0.027366568 | 0.403741082 |
| TNFSF13B | 1.151227806 | 1.402980794 | 0.285321916 | 0.424815448 |
| TNFSF10 | 27.4582505 | 30.36750557 | 0.145288701 | 0.444320553 |
| TNFSF12 | 5.7296537 | 6.817404159 | 0.250774568 | 0.490991226 |
| TNFSF14 | 2.502930883 | 4.406094043 | 0.815881836 | 0.519253253 |
| CD27 | 0.859421548 | 1.740483326 | 1.018050138 | 0.555726 |
| CD80 | 0.105930924 | 0.140947617 | 0.412035279 | 0.588309872 |
| EDA | 0.322674916 | 0.780095396 | 1.273569127 | 0.774172859 |
| TNFRSF10B | 8.6589849 | 9.614607479 | 0.151030053 | 0.785487052 |
| HHLA2 | 0.042029463 | 0.104814418 | 1.318364244 | 0.982324915 |
