# Supplementary material for: Identification and validation a costimulatory molecule gene signature to predict the prognosis and immunotherapy response for hepatocellular carcinoma
Source: Cancer Cell Int. 2022 Feb 22;22:97. doi: 10.1186/s12935-022-02514-0 (PMC8864933; doi:10.1186/s12935-022-02514-0)
Supplement: Supplementary file 2 — Additional file 2: Table S2: The DEGs between high- and low-risk groups in TCGA cohort. [file 12935_2022_2514_MOESM2_ESM.docx]

| Gene | lowMean | highMean | logFC | pValue | fdr |
| --- | --- | --- | --- | --- | --- |
| CCL19 | 29.313768 | 5.108571 | -2.520587 | 4.96E-05 | 0.0001423 |
| IGLC7 | 3.7011369 | 0.697228 | -2.408266 | 0.0026198 | 0.0049194 |
| IGLV1-36 | 1.6425588 | 0.3914657 | -2.068987 | 0.0022089 | 0.004227 |
| CCL21 | 55.358799 | 13.242028 | -2.063689 | 0.0021365 | 0.0041018 |
| MIR8071-2 | 1.6660189 | 0.4407315 | -1.918433 | 0.0010942 | 0.0022566 |
| FAM83A-AS1 | 10.80787 | 2.9555286 | -1.870594 | 3.59E-05 | 0.0001072 |
| IGHV4-28 | 2.5443901 | 0.6966465 | -1.868821 | 0.0223405 | 0.0336017 |
| IGLV4-60 | 2.2032792 | 0.6089805 | -1.855184 | 0.0028708 | 0.0053393 |
| IGKV1-9 | 21.409475 | 6.6392995 | -1.689146 | 0.0119197 | 0.019064 |
| PLAC9 | 6.2605498 | 2.1126374 | -1.567244 | 0.0003423 | 0.0008019 |
| CD79A | 3.661391 | 1.2652239 | -1.532999 | 0.0015567 | 0.0031001 |
| PFN1P11 | 6.5246634 | 2.2802973 | -1.516682 | 0.000169 | 0.000429 |
| IGKV1-17 | 11.001146 | 3.8578384 | -1.511789 | 0.0059945 | 0.0103365 |
| IGHM | 40.833039 | 14.350954 | -1.50859 | 0.0046908 | 0.00831 |
| IGHD | 1.9867626 | 0.7043049 | -1.496147 | 0.0175194 | 0.0269739 |
| IGHG2 | 100.26876 | 35.663117 | -1.491367 | 0.0137232 | 0.0216383 |
| IGHGP | 12.266413 | 4.4737735 | -1.455149 | 0.0173401 | 0.0267226 |
| IL7R | 2.3657736 | 0.8764663 | -1.432541 | 4.86E-06 | 1.85E-05 |
| IGHV3-72 | 1.4894706 | 0.5518833 | -1.432364 | 0.0227391 | 0.0341482 |
| IGKV1-27 | 7.5033859 | 2.7957173 | -1.424323 | 0.0090554 | 0.0149017 |
| CTH | 43.17236 | 16.498031 | -1.387814 | 6.33E-06 | 2.34E-05 |
| COX7A1 | 10.065402 | 3.8687095 | -1.379481 | 0.0012937 | 0.0026313 |
| IGLV2-14 | 33.142496 | 12.980766 | -1.352307 | 0.0347539 | 0.0498637 |
| PLA2G2D | 1.6635605 | 0.6524799 | -1.350269 | 0.0041982 | 0.0075288 |
| PLA2G2A | 380.51034 | 149.71087 | -1.345757 | 0.0004424 | 0.0010097 |
| ACKR1 | 1.6470688 | 0.6525747 | -1.335686 | 2.44E-07 | 1.37E-06 |
| FABP4 | 13.762506 | 5.5872547 | -1.300532 | 6.76E-07 | 3.30E-06 |
| IGHV3-20 | 1.5775599 | 0.6448261 | -1.290713 | 0.0073163 | 0.0123307 |
| AQP8 | 4.6366104 | 1.9277564 | -1.266148 | 0.0021621 | 0.0041462 |
| IGKV2-24 | 3.4969277 | 1.4578809 | -1.262215 | 0.0196683 | 0.0299186 |
| NNMT | 199.44641 | 83.226777 | -1.260881 | 3.89E-05 | 0.000115 |
| IGHA1 | 134.23991 | 56.056135 | -1.259869 | 0.0068497 | 0.0116352 |
| AL354872.1 | 12.659923 | 5.3452418 | -1.243942 | 4.69E-09 | 4.94E-08 |
| IGHV3-11 | 11.594747 | 4.8981646 | -1.243158 | 0.0340827 | 0.0490578 |
| IGLV6-57 | 6.2628762 | 2.6579538 | -1.236509 | 0.0182389 | 0.0279635 |
| GNMT | 97.617664 | 41.708013 | -1.226818 | 4.05E-07 | 2.11E-06 |
| IGHA2 | 14.124493 | 6.0544923 | -1.222121 | 0.0333723 | 0.0481246 |
| IGLV3-1 | 14.973498 | 6.4456769 | -1.216008 | 0.0161856 | 0.0251096 |
| SAA1 | 1727.6354 | 744.98216 | -1.213521 | 0.0006688 | 0.0014555 |
| IGLV2-11 | 22.252765 | 9.6297453 | -1.208415 | 0.005286 | 0.0092406 |
| STEAP4 | 2.0247734 | 0.8765131 | -1.207913 | 5.54E-08 | 3.90E-07 |
| KLRB1 | 3.0671353 | 1.3293435 | -1.206178 | 2.19E-11 | 6.34E-10 |
| CLEC3B | 10.526514 | 4.5816992 | -1.200073 | 1.84E-16 | 1.65E-13 |
| IGLC3 | 76.541016 | 33.880808 | -1.175765 | 0.0040944 | 0.0073653 |
| UPP2 | 4.9226195 | 2.2110357 | -1.154704 | 1.07E-07 | 6.84E-07 |
| SAA2 | 204.80647 | 93.026033 | -1.138555 | 0.0010576 | 0.0021898 |
| MT1M | 41.584338 | 18.969974 | -1.132323 | 0.0026733 | 0.0050061 |
| CYP2E1 | 364.72664 | 166.40213 | -1.132142 | 1.19E-09 | 1.56E-08 |
| SPDYC | 3.7075167 | 1.6929148 | -1.130944 | 0.0008008 | 0.0017085 |
| IGHG3 | 47.942632 | 22.071046 | -1.119154 | 0.0295779 | 0.0431552 |
| FBLN2 | 6.9154647 | 3.1885782 | -1.116913 | 0.0005817 | 0.0012855 |
| DNASE1L3 | 6.548621 | 3.0234535 | -1.114994 | 6.50E-13 | 4.32E-11 |
| AC079360.1 | 1.9435606 | 0.8976236 | -1.11452 | 7.63E-06 | 2.75E-05 |
| MT2A | 486.34455 | 226.61947 | -1.101707 | 7.08E-05 | 0.0001956 |
| TTC36 | 16.804559 | 7.9282262 | -1.083783 | 4.24E-06 | 1.64E-05 |
| MT1X | 126.41584 | 59.677581 | -1.082916 | 0.0001398 | 0.0003623 |
| HPD | 723.88202 | 344.32451 | -1.071986 | 2.97E-10 | 5.06E-09 |
| IGKC | 250.02947 | 118.95687 | -1.07166 | 0.0211941 | 0.0320097 |
| MZB1 | 3.6623655 | 1.744133 | -1.070266 | 0.0219692 | 0.0330904 |
| AC012065.3 | 14.656677 | 6.9875956 | -1.06869 | 1.27E-05 | 4.31E-05 |
| TAT | 206.10022 | 98.688888 | -1.062386 | 2.17E-08 | 1.75E-07 |
| CFHR3 | 37.214135 | 17.990937 | -1.04858 | 4.17E-08 | 3.06E-07 |
| MT1G | 426.82064 | 212.03241 | -1.009345 | 0.022538 | 0.0338681 |
| SLC39A10 | 1.0764059 | 2.1529525 | 1.0000943 | 2.71E-13 | 2.12E-11 |
| SLC29A4 | 3.2143632 | 6.4381645 | 1.0021165 | 0.0002349 | 0.0005764 |
| MAGEA3 | 3.8958328 | 7.8216839 | 1.0055475 | 0.006958 | 0.0118021 |
| AL390728.4 | 2.5667783 | 5.1556747 | 1.0062026 | 8.71E-11 | 1.94E-09 |
| LMNB2 | 3.6858755 | 7.4067573 | 1.0068347 | 3.35E-10 | 5.60E-09 |
| CARMIL1 | 0.9209265 | 1.8511578 | 1.00727 | 6.35E-09 | 6.31E-08 |
| LPCAT1 | 4.6212172 | 9.3153132 | 1.0113314 | 1.10E-09 | 1.46E-08 |
| HMMR | 1.635896 | 3.3004615 | 1.0125868 | 1.31E-11 | 4.26E-10 |
| CDH24 | 0.6653417 | 1.3440742 | 1.0144454 | 1.32E-06 | 5.91E-06 |
| TACC3 | 3.1450597 | 6.3561325 | 1.0150618 | 1.43E-10 | 2.84E-09 |
| CDC7 | 0.9000678 | 1.8207869 | 1.0164564 | 1.32E-10 | 2.67E-09 |
| HROB | 0.7482647 | 1.5145693 | 1.0172869 | 6.21E-12 | 2.42E-10 |
| ITGAV | 5.9891302 | 12.158488 | 1.0215454 | 1.05E-07 | 6.77E-07 |
| TMC6 | 1.8438713 | 3.7501807 | 1.0242221 | 0.0049537 | 0.0087225 |
| LAMA5 | 3.4444638 | 7.0072681 | 1.0245727 | 7.89E-08 | 5.27E-07 |
| SUSD4 | 2.5997885 | 5.2925955 | 1.0255812 | 1.16E-06 | 5.27E-06 |
| MAD2L1 | 1.0318449 | 2.1047763 | 1.0284407 | 1.89E-10 | 3.54E-09 |
| TINAG | 1.6194876 | 3.3037486 | 1.0285665 | 0.0006837 | 0.0014836 |
| TEDC2 | 1.231035 | 2.5117113 | 1.0287989 | 8.25E-11 | 1.87E-09 |
| SLC7A1 | 0.9476953 | 1.934024 | 1.0291106 | 0.0001077 | 0.0002861 |
| P3H4 | 2.6714684 | 5.4579147 | 1.0307169 | 3.70E-07 | 1.95E-06 |
| HID1 | 1.7604192 | 3.6003786 | 1.0322296 | 0.0005472 | 0.0012166 |
| PLXNA3 | 0.9370689 | 1.9180177 | 1.033389 | 1.40E-07 | 8.61E-07 |
| FAM117B | 0.788282 | 1.6155369 | 1.03523 | 2.76E-07 | 1.52E-06 |
| PLP2 | 21.933519 | 44.951768 | 1.0352406 | 0.0005923 | 0.0013059 |
| OIP5 | 1.2657829 | 2.6012748 | 1.0391888 | 1.58E-10 | 3.07E-09 |
| CDCA5 | 2.3001692 | 4.7338715 | 1.0412806 | 5.53E-12 | 2.21E-10 |
| PYCR1 | 5.3262342 | 10.979701 | 1.043651 | 0.0004304 | 0.0009864 |
| FMNL2 | 1.4960345 | 3.0864625 | 1.0448108 | 2.34E-06 | 9.72E-06 |
| MIR3189 | 0.9838054 | 2.0316244 | 1.0461889 | 0.0105822 | 0.0171596 |
| TRAIP | 0.8344321 | 1.7245784 | 1.0473772 | 2.27E-13 | 1.93E-11 |
| MFSD10 | 5.9318507 | 12.272603 | 1.0488871 | 6.38E-06 | 2.35E-05 |
| GRB7 | 2.9692988 | 6.1464589 | 1.0496332 | 2.12E-06 | 8.90E-06 |
| CTHRC1 | 3.2180803 | 6.667093 | 1.0508575 | 0.0031576 | 0.0058084 |
| ZWINT | 5.1291939 | 10.638908 | 1.0525461 | 2.37E-12 | 1.14E-10 |
| BTNL8 | 0.9894391 | 2.0532145 | 1.0532014 | 0.0048044 | 0.0084882 |
| ARNTL2 | 1.0359514 | 2.1511613 | 1.0541594 | 1.59E-07 | 9.61E-07 |
| AGRN | 11.080392 | 23.03043 | 1.0555324 | 2.78E-10 | 4.80E-09 |
| AC009005.1 | 0.9065211 | 1.8850252 | 1.0561713 | 8.27E-10 | 1.15E-08 |
| NCAPD2 | 3.2789724 | 6.821998 | 1.0569506 | 2.33E-13 | 1.95E-11 |
| AKR1B1 | 5.1900105 | 10.814613 | 1.0591727 | 0.0019369 | 0.0037615 |
| TLDC2 | 0.7913259 | 1.6498967 | 1.0600318 | 4.80E-06 | 1.83E-05 |
| AL138826.1 | 5.1766206 | 10.798784 | 1.0607864 | 0.0290537 | 0.0424915 |
| NRM | 4.7022445 | 9.8156978 | 1.0617413 | 6.12E-10 | 9.02E-09 |
| SPC25 | 1.2392721 | 2.5878957 | 1.0622865 | 1.49E-12 | 8.20E-11 |
| SULT1C2 | 1.4968086 | 3.1310974 | 1.0647786 | 3.52E-07 | 1.87E-06 |
| AP000769.1 | 0.9792432 | 2.0487141 | 1.0649796 | 1.40E-06 | 6.22E-06 |
| CDKN2A | 2.6449422 | 5.546322 | 1.0682952 | 3.21E-06 | 1.28E-05 |
| ZBTB12 | 1.2559696 | 2.6374231 | 1.0703274 | 1.94E-12 | 9.97E-11 |
| CHML | 1.3918097 | 2.9244156 | 1.0711864 | 1.08E-11 | 3.66E-10 |
| PKMYT1 | 0.8642499 | 1.8170836 | 1.0721042 | 4.66E-12 | 1.97E-10 |
| MPZ | 4.8117341 | 10.120725 | 1.0726838 | 0.0279105 | 0.0410048 |
| PKIB | 1.6160555 | 3.4055245 | 1.0754002 | 0.0004838 | 0.0010928 |
| RAD51 | 0.7724957 | 1.6280887 | 1.0755804 | 6.30E-11 | 1.48E-09 |
| SOX9 | 6.4307515 | 13.568624 | 1.0772152 | 1.20E-07 | 7.54E-07 |
| CD24 | 30.508728 | 64.460593 | 1.0791954 | 2.45E-06 | 1.01E-05 |
| CDC25A | 0.7188942 | 1.5220701 | 1.0821834 | 2.89E-09 | 3.31E-08 |
| PRC1 | 2.4084213 | 5.1045098 | 1.0836846 | 1.82E-13 | 1.64E-11 |
| EPCAM | 13.990811 | 29.6591 | 1.0839952 | 0.0099744 | 0.0162556 |
| FANCI | 1.1695428 | 2.4794302 | 1.0840639 | 5.86E-15 | 1.76E-12 |
| CDC45 | 1.5080312 | 3.2012852 | 1.0859849 | 1.29E-11 | 4.22E-10 |
| MIR4292 | 1.298283 | 2.7569314 | 1.0864585 | 4.00E-10 | 6.42E-09 |
| SCGN | 6.316788 | 13.431726 | 1.0883817 | 0.0047842 | 0.0084612 |
| LPCAT4 | 1.0648364 | 2.2658877 | 1.0894446 | 1.25E-06 | 5.61E-06 |
| RACGAP1 | 2.3733756 | 5.0550385 | 1.0907817 | 2.18E-15 | 9.78E-13 |
| FUT4 | 0.7520513 | 1.602021 | 1.09099 | 0.0003061 | 0.0007277 |
| SINHCAF | 1.472697 | 3.1416034 | 1.0930405 | 4.14E-07 | 2.15E-06 |
| SMC4 | 1.5326725 | 3.2721145 | 1.0941737 | 5.55E-13 | 3.87E-11 |
| NCK2 | 5.0969254 | 10.89208 | 1.0955804 | 1.77E-07 | 1.05E-06 |
| CREB3L1 | 1.4144596 | 3.023806 | 1.0961146 | 0.004777 | 0.0084497 |
| CHST11 | 1.2259868 | 2.6264849 | 1.0991898 | 1.74E-05 | 5.63E-05 |
| CLGN | 2.9429032 | 6.3096597 | 1.1003221 | 7.22E-06 | 2.62E-05 |
| HOMER3 | 2.5698636 | 5.5115644 | 1.1007701 | 9.53E-07 | 4.44E-06 |
| S100A6 | 64.316122 | 138.04963 | 1.1019347 | 0.01658 | 0.0256667 |
| NDC80 | 1.5416531 | 3.312209 | 1.1033155 | 8.97E-12 | 3.17E-10 |
| TSPAN1 | 1.5106885 | 3.250267 | 1.105352 | 0.0257061 | 0.0380784 |
| ZFPM2-AS1 | 0.9407913 | 2.0274131 | 1.1076935 | 0.0227645 | 0.034182 |
| MAGEA6 | 3.000599 | 6.4749944 | 1.1096284 | 0.0005654 | 0.0012536 |
| MAFG-DT | 0.9258222 | 2.0010424 | 1.1119446 | 4.92E-07 | 2.51E-06 |
| COL4A5 | 0.669914 | 1.4486138 | 1.1126252 | 0.0001707 | 0.000433 |
| PSRC1 | 1.2555914 | 2.7253134 | 1.1180552 | 4.23E-13 | 3.04E-11 |
| DTL | 1.5948851 | 3.4679779 | 1.1206422 | 2.47E-14 | 4.12E-12 |
| PODXL2 | 3.1776538 | 6.909848 | 1.120692 | 0.0011762 | 0.0024121 |
| NFE2L3 | 1.3265874 | 2.8959672 | 1.1263255 | 3.72E-07 | 1.96E-06 |
| HHIPL2 | 1.0128716 | 2.2142598 | 1.1283732 | 0.0328875 | 0.0474838 |
| IL4I1 | 1.188925 | 2.6004651 | 1.1291119 | 0.0073674 | 0.0124044 |
| SNORA73B | 0.709181 | 1.551894 | 1.1298042 | 0.0037074 | 0.006742 |
| PPM1H | 1.0999542 | 2.4107233 | 1.1320226 | 5.28E-07 | 2.66E-06 |
| PLAU | 2.5034729 | 5.4928157 | 1.133615 | 0.0328913 | 0.0474838 |
| SMARCD3 | 1.1293559 | 2.4801374 | 1.1349199 | 0.0036236 | 0.0066042 |
| KCNK5 | 2.6403967 | 5.8012992 | 1.1356213 | 0.0007412 | 0.0015952 |
| AURKB | 2.6202045 | 5.7594451 | 1.1362504 | 5.05E-08 | 3.60E-07 |
| CCNB1 | 5.8726289 | 12.923513 | 1.1379199 | 8.10E-13 | 5.07E-11 |
| FANCD2 | 0.6318264 | 1.3924907 | 1.1400676 | 8.85E-14 | 9.82E-12 |
| ASPM | 1.2639458 | 2.7890064 | 1.1418166 | 2.06E-12 | 1.04E-10 |
| DDX11 | 0.9586236 | 2.1154798 | 1.1419486 | 2.97E-17 | 1.15E-13 |
| ADAM9 | 3.4407701 | 7.5958749 | 1.1424846 | 1.51E-08 | 1.30E-07 |
| LIMCH1 | 0.8506504 | 1.8791696 | 1.143457 | 0.002068 | 0.0039874 |
| CDCA3 | 0.9206199 | 2.0387138 | 1.1469817 | 6.84E-12 | 2.63E-10 |
| CCNB2 | 2.5159677 | 5.5779252 | 1.1486152 | 1.68E-11 | 5.10E-10 |
| SKA3 | 0.9616798 | 2.1344879 | 1.1502615 | 4.73E-12 | 1.97E-10 |
| CRACR2B | 1.293911 | 2.8738122 | 1.1512275 | 0.0046624 | 0.0082684 |
| NCS1 | 1.2053926 | 2.6822613 | 1.1539467 | 2.59E-07 | 1.44E-06 |
| CD109 | 0.9598922 | 2.1359856 | 1.1539576 | 1.04E-07 | 6.70E-07 |
| CENPM | 2.6218794 | 5.8401874 | 1.1554133 | 9.12E-11 | 2.00E-09 |
| USH1C | 2.0947907 | 4.6753839 | 1.1582787 | 1.41E-06 | 6.24E-06 |
| RAD51AP1 | 1.0890825 | 2.4310528 | 1.158468 | 2.50E-13 | 2.01E-11 |
| ARHGAP11A | 0.8885854 | 1.9836669 | 1.1585874 | 3.00E-13 | 2.31E-11 |
| SERPINE2 | 2.0721402 | 4.6373206 | 1.1621698 | 0.0078292 | 0.013108 |
| RASSF8 | 0.7638386 | 1.7134159 | 1.1655358 | 1.26E-07 | 7.89E-07 |
| RASEF | 0.9663762 | 2.1705683 | 1.1674161 | 7.70E-06 | 2.77E-05 |
| PCLAF | 1.6630799 | 3.7396418 | 1.1690426 | 1.67E-12 | 8.90E-11 |
| EHF | 1.2683444 | 2.8652493 | 1.1757141 | 6.35E-06 | 2.34E-05 |
| MAFA-AS1 | 1.0897141 | 2.4626448 | 1.1762589 | 0.0110706 | 0.0178596 |
| NDRG1 | 15.727923 | 35.544171 | 1.1762848 | 1.02E-06 | 4.71E-06 |
| RHPN1 | 1.1778377 | 2.665428 | 1.1782265 | 3.15E-06 | 1.26E-05 |
| SLC2A5 | 0.9423045 | 2.1332149 | 1.178764 | 0.0038914 | 0.0070339 |
| LINC00221 | 0.6809096 | 1.543532 | 1.1807003 | 0.0075631 | 0.0127063 |
| SERPINA12 | 1.1168626 | 2.5339696 | 1.1819475 | 0.0018556 | 0.0036222 |
| ASF1B | 2.7709192 | 6.2873362 | 1.1820843 | 1.75E-11 | 5.27E-10 |
| NDUFA4L2 | 6.5816771 | 14.94154 | 1.1828017 | 0.0027909 | 0.0052037 |
| MEP1A | 2.1549064 | 4.8979112 | 1.1845414 | 0.0001436 | 0.0003715 |
| ELF4 | 0.9549299 | 2.171836 | 1.1854485 | 0.0001607 | 0.0004107 |
| KRT20 | 2.6229231 | 5.9738057 | 1.1874748 | 0.0139279 | 0.0219276 |
| MAGEA12 | 1.8546834 | 4.2365346 | 1.1917117 | 0.0215937 | 0.0325752 |
| HNF1B | 2.5393861 | 5.800646 | 1.1917338 | 0.0010834 | 0.0022355 |
| DLG3 | 0.6447562 | 1.4750429 | 1.1939313 | 0.0001064 | 0.000283 |
| BIRC5 | 4.2497852 | 9.7268713 | 1.1945859 | 4.07E-11 | 1.05E-09 |
| CDCA7L | 1.0703126 | 2.4517243 | 1.1957645 | 1.06E-09 | 1.41E-08 |
| PFN2 | 2.8596558 | 6.557452 | 1.1972938 | 0.020762 | 0.0314181 |
| PAQR4 | 1.6347077 | 3.7490844 | 1.1975056 | 1.25E-12 | 7.09E-11 |
| CDS1 | 0.832842 | 1.910115 | 1.1975447 | 0.0003225 | 0.0007623 |
| LINC01287 | 1.784203 | 4.0940071 | 1.1982339 | 0.0021184 | 0.0040719 |
| PAFAH1B3 | 8.466281 | 19.486662 | 1.2026867 | 2.33E-08 | 1.85E-07 |
| MCM2 | 4.6122957 | 10.617065 | 1.202828 | 3.27E-14 | 4.76E-12 |
| FMO1 | 0.9871054 | 2.2734595 | 1.2036133 | 0.0057721 | 0.009987 |
| SFN | 15.435757 | 35.575829 | 1.2046211 | 1.34E-05 | 4.48E-05 |
| KIFC1 | 3.1134693 | 7.1770946 | 1.2048769 | 1.60E-12 | 8.61E-11 |
| INAVA | 0.9201992 | 2.1231766 | 1.2062062 | 5.02E-08 | 3.59E-07 |
| MKI67 | 1.6860876 | 3.8957831 | 1.2082339 | 2.01E-11 | 5.87E-10 |
| ETV4 | 3.9424835 | 9.1155866 | 1.2092308 | 8.13E-07 | 3.86E-06 |
| SPINT1-AS1 | 0.6771434 | 1.5702674 | 1.213477 | 0.0002814 | 0.0006751 |
| STK39 | 1.8612201 | 4.3190194 | 1.2144551 | 1.03E-09 | 1.38E-08 |
| KIF11 | 1.1743928 | 2.7256841 | 1.2147034 | 3.31E-13 | 2.51E-11 |
| KCNJ11 | 0.6466392 | 1.5042251 | 1.2179876 | 3.30E-09 | 3.69E-08 |
| AC092868.2 | 0.667551 | 1.5529029 | 1.2180176 | 4.02E-06 | 1.57E-05 |
| TOR4A | 1.2247589 | 2.8492026 | 1.2180605 | 3.62E-05 | 0.0001079 |
| GLIS2 | 1.8041387 | 4.1991016 | 1.2187704 | 4.42E-09 | 4.70E-08 |
| AKR1B15 | 1.2077307 | 2.8142692 | 1.2204615 | 0.0251438 | 0.0373501 |
| PAGE1 | 6.2566449 | 14.583625 | 1.2208882 | 0.0013953 | 0.0028143 |
| ASNS | 1.5530014 | 3.6202135 | 1.2210156 | 1.10E-06 | 5.02E-06 |
| IMPDH1 | 2.5538274 | 5.9569984 | 1.2219245 | 1.47E-05 | 4.87E-05 |
| NEK2 | 1.7636594 | 4.1170832 | 1.2230506 | 7.87E-14 | 8.90E-12 |
| ZNF320 | 0.6278716 | 1.4675698 | 1.2248877 | 2.83E-07 | 1.55E-06 |
| UAP1L1 | 1.1621395 | 2.7164417 | 1.2249348 | 0.0023396 | 0.0044499 |
| HILPDA | 1.9931737 | 4.6596221 | 1.2251456 | 1.22E-10 | 2.50E-09 |
| KNTC1 | 0.7436367 | 1.7418835 | 1.2279783 | 2.83E-17 | 1.15E-13 |
| IGF2BP1 | 1.119471 | 2.6226837 | 1.2282266 | 1.67E-06 | 7.25E-06 |
| B3GNT5 | 0.8648315 | 2.027703 | 1.2293553 | 1.08E-08 | 9.86E-08 |
| AKR1B10P1 | 1.4196576 | 3.3316115 | 1.2306772 | 0.0265401 | 0.0391943 |
| TMPRSS3 | 1.1376902 | 2.6730361 | 1.2323715 | 9.78E-05 | 0.0002618 |
| EXO1 | 0.7693759 | 1.8101442 | 1.2343441 | 9.53E-14 | 1.02E-11 |
| NCAPG | 1.3325373 | 3.1376924 | 1.235528 | 7.60E-13 | 4.86E-11 |
| SLC25A24 | 0.6369202 | 1.4997948 | 1.2355806 | 1.55E-05 | 5.12E-05 |
| PLAUR | 1.105468 | 2.6123513 | 1.2406916 | 0.000313 | 0.0007421 |
| ANXA13 | 5.0232697 | 11.879776 | 1.241809 | 0.0001352 | 0.0003516 |
| NRSN2 | 3.0850581 | 7.3145794 | 1.2454773 | 1.32E-08 | 1.16E-07 |
| FBLN1 | 4.5707894 | 10.850127 | 1.2471966 | 0.0168457 | 0.0260469 |
| CDK1 | 2.5459738 | 6.0469611 | 1.2479927 | 1.11E-12 | 6.47E-11 |
| AC022784.1 | 0.603309 | 1.4334968 | 1.2485697 | 0.000874 | 0.0018489 |
| TPX2 | 5.4341647 | 12.93373 | 1.2510082 | 1.22E-14 | 2.69E-12 |
| ENTPD2 | 1.1651896 | 2.7808197 | 1.2549454 | 0.0002904 | 0.0006948 |
| ADGRG1 | 2.0483806 | 4.8959191 | 1.2570959 | 0.0046483 | 0.0082471 |
| LAMB3 | 4.0588781 | 9.7030525 | 1.2573577 | 0.0002926 | 0.0006995 |
| SLC2A1 | 1.4970246 | 3.5927811 | 1.2630031 | 1.89E-06 | 8.04E-06 |
| CKAP2L | 0.6186399 | 1.4848718 | 1.2631666 | 1.20E-14 | 2.69E-12 |
| BUB1 | 0.8901672 | 2.1370818 | 1.2634939 | 2.00E-14 | 3.67E-12 |
| SPP1 | 199.12882 | 478.76404 | 1.2656127 | 3.73E-05 | 0.0001108 |
| PBK | 1.8025889 | 4.3351611 | 1.2660152 | 1.01E-11 | 3.44E-10 |
| PLCD3 | 0.7224151 | 1.7400374 | 1.2682184 | 1.87E-06 | 7.98E-06 |
| CCNE1 | 2.0433122 | 4.9229389 | 1.2686101 | 7.01E-09 | 6.84E-08 |
| TYRO3 | 0.6283403 | 1.5142492 | 1.2689847 | 3.47E-12 | 1.55E-10 |
| MELK | 1.4938009 | 3.6016859 | 1.2696845 | 1.39E-13 | 1.32E-11 |
| C2CD4A | 1.0277865 | 2.4808006 | 1.2712652 | 0.0051824 | 0.0090786 |
| CCL28 | 0.9039496 | 2.1840367 | 1.2726828 | 4.15E-08 | 3.05E-07 |
| BAIAP2L2 | 3.988657 | 9.6796843 | 1.2790569 | 2.51E-05 | 7.81E-05 |
| TROAP | 1.5079306 | 3.6653747 | 1.2813907 | 9.66E-12 | 3.34E-10 |
| MAP3K21 | 0.7099848 | 1.726937 | 1.2823554 | 5.70E-08 | 3.99E-07 |
| GINS1 | 1.4342303 | 3.4920498 | 1.2837975 | 4.17E-16 | 2.95E-13 |
| PLBD1 | 1.9974411 | 4.886185 | 1.2905556 | 0.0005551 | 0.0012329 |
| THSD4 | 0.6976858 | 1.7067927 | 1.2906386 | 0.0017546 | 0.0034488 |
| FOXN4 | 0.6572019 | 1.609868 | 1.2925338 | 0.0082563 | 0.0137282 |
| CYP2S1 | 0.7217805 | 1.7785725 | 1.3010877 | 0.0016475 | 0.0032613 |
| TMEM51 | 2.2128482 | 5.4622851 | 1.3036001 | 1.98E-05 | 6.34E-05 |
| EPS8L3 | 3.285968 | 8.130619 | 1.3070467 | 3.46E-07 | 1.84E-06 |
| ASRGL1 | 0.9829237 | 2.4341791 | 1.308284 | 5.04E-07 | 2.56E-06 |
| DLGAP5 | 1.0844759 | 2.6872324 | 1.3091232 | 4.17E-13 | 3.04E-11 |
| TRIM45 | 0.6181367 | 1.5375476 | 1.3146332 | 6.78E-15 | 1.90E-12 |
| VLDLR | 0.5867654 | 1.4599715 | 1.3150846 | 0.0010797 | 0.0022295 |
| TOP2A | 4.465341 | 11.111818 | 1.3152526 | 1.27E-13 | 1.24E-11 |
| FZD7 | 0.6548029 | 1.6310644 | 1.3166812 | 0.0002127 | 0.0005289 |
| MEX3A | 0.7031384 | 1.7543301 | 1.3190396 | 2.38E-13 | 1.95E-11 |
| SLC6A8 | 3.5815863 | 8.9503702 | 1.3213486 | 4.49E-06 | 1.73E-05 |
| SOX4 | 4.1146096 | 10.303593 | 1.3243201 | 1.76E-10 | 3.35E-09 |
| MUC3A | 1.1707567 | 2.9334076 | 1.3251362 | 6.55E-05 | 0.0001822 |
| LINC00665 | 0.8018638 | 2.0092699 | 1.3252422 | 1.26E-05 | 4.26E-05 |
| DBN1 | 3.5281645 | 8.8529223 | 1.3272359 | 0.0003593 | 0.0008382 |
| CDC6 | 1.6630412 | 4.1747933 | 1.3278809 | 4.64E-14 | 6.08E-12 |
| PRR11 | 0.9569098 | 2.4038261 | 1.3288778 | 1.25E-14 | 2.70E-12 |
| STC2 | 1.1224918 | 2.8212723 | 1.3296411 | 7.91E-06 | 2.84E-05 |
| DKK1 | 5.1987085 | 13.086224 | 1.3318237 | 0.0010349 | 0.0021458 |
| RAB25 | 2.4303669 | 6.1355286 | 1.3360135 | 0.0160727 | 0.0249543 |
| FOXM1 | 2.2464559 | 5.6983196 | 1.3428858 | 2.57E-14 | 4.21E-12 |
| KIF20A | 1.6752124 | 4.2617264 | 1.347094 | 1.31E-14 | 2.77E-12 |
| HJURP | 1.3481855 | 3.4316204 | 1.347871 | 3.05E-14 | 4.56E-12 |
| UBE2C | 7.6136376 | 19.461612 | 1.3539734 | 1.77E-09 | 2.18E-08 |
| RAP1GAP | 5.5097446 | 14.094788 | 1.3551044 | 8.88E-11 | 1.97E-09 |
| UHRF1 | 0.7828506 | 2.0042205 | 1.3562323 | 9.47E-12 | 3.29E-10 |
| CCDC9B | 0.8133461 | 2.0831573 | 1.3568306 | 1.94E-05 | 6.21E-05 |
| CENPA | 1.0206967 | 2.6171404 | 1.3584371 | 1.78E-12 | 9.26E-11 |
| NCAPH | 1.1407997 | 2.9341974 | 1.3629204 | 1.12E-15 | 5.67E-13 |
| TNFSF4 | 0.574521 | 1.4798702 | 1.3650392 | 3.26E-12 | 1.48E-10 |
| CENPF | 1.3145561 | 3.3919945 | 1.3675581 | 2.66E-15 | 1.06E-12 |
| TTK | 0.7254582 | 1.8726406 | 1.3681096 | 8.57E-13 | 5.34E-11 |
| CHAF1B | 0.7957958 | 2.0553231 | 1.3688951 | 1.31E-13 | 1.26E-11 |
| ITGB1-DT | 0.8722425 | 2.2548203 | 1.3702112 | 0.0132286 | 0.0209294 |
| ECT2 | 1.5230913 | 3.9408259 | 1.3714956 | 3.49E-16 | 2.71E-13 |
| TSPAN15 | 4.2309757 | 10.95469 | 1.3724863 | 4.80E-05 | 0.0001383 |
| SEL1L3 | 3.2420697 | 8.4097149 | 1.3751418 | 0.0014608 | 0.0029306 |
| CDCA8 | 2.1687301 | 5.6438473 | 1.3798285 | 1.63E-16 | 1.58E-13 |
| CDR2L | 1.0520356 | 2.7422167 | 1.382159 | 0.0051362 | 0.0090003 |
| ABCC1 | 1.3260244 | 3.4622198 | 1.38459 | 1.52E-09 | 1.92E-08 |
| NRCAM | 0.8280548 | 2.1640529 | 1.3859377 | 1.68E-07 | 1.00E-06 |
| NT5DC2 | 3.4582985 | 9.0576823 | 1.3890795 | 2.13E-08 | 1.72E-07 |
| SHCBP1 | 0.5699423 | 1.4934312 | 1.389743 | 5.68E-10 | 8.54E-09 |
| ORC1 | 0.7806382 | 2.0461976 | 1.3902195 | 2.08E-15 | 9.71E-13 |
| ENO2 | 0.8925316 | 2.3444139 | 1.3932521 | 2.30E-05 | 7.25E-05 |
| SLC38A1 | 2.4172437 | 6.3504998 | 1.3935072 | 3.87E-09 | 4.24E-08 |
| MIR210HG | 0.7780319 | 2.0669063 | 1.4095718 | 5.17E-12 | 2.10E-10 |
| PLK1 | 1.4979827 | 4.0057935 | 1.4190671 | 7.16E-15 | 1.94E-12 |
| MLLT11 | 0.9250264 | 2.4764852 | 1.4207275 | 5.12E-07 | 2.59E-06 |
| S100A11 | 63.270488 | 169.54811 | 1.4220901 | 0.0007154 | 0.0015435 |
| AL355102.4 | 1.3744979 | 3.6851413 | 1.4228152 | 0.0012172 | 0.0024896 |
| KIF4A | 1.521205 | 4.07917 | 1.4230611 | 5.00E-14 | 6.36E-12 |
| SKA1 | 1.0908928 | 2.9339944 | 1.4273568 | 1.78E-13 | 1.64E-11 |
| TMEM54 | 5.5963138 | 15.117455 | 1.4336665 | 1.89E-07 | 1.11E-06 |
| MAGEB2 | 1.2121388 | 3.2744283 | 1.4336881 | 0.0022728 | 0.0043377 |
| CXCL1 | 4.7246185 | 12.782425 | 1.4358918 | 0.0001094 | 0.0002904 |
| COLCA2 | 0.8516945 | 2.3134989 | 1.4416685 | 8.51E-09 | 8.09E-08 |
| BUB1B | 0.8702346 | 2.3670585 | 1.443619 | 3.38E-15 | 1.27E-12 |
| NUF2 | 1.1897956 | 3.2407341 | 1.4456069 | 6.00E-15 | 1.76E-12 |
| MSC | 4.898838 | 13.354231 | 1.4467854 | 0.0258352 | 0.0382406 |
| CDC20 | 5.6476895 | 15.489346 | 1.4555435 | 5.35E-12 | 2.16E-10 |
| MFAP2 | 0.6185077 | 1.6964659 | 1.4556689 | 0.0013292 | 0.0026958 |
| KEL | 0.5988069 | 1.6462338 | 1.4590065 | 0.0061973 | 0.0106431 |
| LIF | 0.9888679 | 2.7281674 | 1.4640824 | 0.0007078 | 0.0015297 |
| SLC6A6 | 0.857884 | 2.3679623 | 1.4647916 | 0.0003999 | 0.0009232 |
| CABYR | 1.1345263 | 3.1402534 | 1.468791 | 0.0024634 | 0.0046551 |
| PI3 | 3.529071 | 9.7957859 | 1.4728728 | 0.0024051 | 0.0045597 |
| PAQR5 | 1.180452 | 3.2766704 | 1.4728912 | 4.17E-08 | 3.06E-07 |
| DMKN | 0.8834165 | 2.4572395 | 1.4758728 | 0.0019946 | 0.0038593 |
| PFKFB3 | 3.6741681 | 10.257172 | 1.4811435 | 2.94E-06 | 1.19E-05 |
| GTSE1 | 0.7508781 | 2.09759 | 1.4820822 | 9.01E-15 | 2.33E-12 |
| KIF12 | 5.2246298 | 14.646386 | 1.487144 | 1.16E-11 | 3.90E-10 |
| KIF18B | 0.8251467 | 2.3223781 | 1.4928804 | 1.91E-14 | 3.65E-12 |
| TTC39A | 0.774368 | 2.1892888 | 1.499371 | 6.81E-06 | 2.49E-05 |
| AC099850.4 | 1.0751933 | 3.0417935 | 1.5003262 | 2.64E-15 | 1.06E-12 |
| DUSP9 | 5.7527553 | 16.396137 | 1.5110309 | 0.0003015 | 0.0007182 |
| CTAG2 | 3.1180896 | 8.8993314 | 1.5130346 | 3.53E-05 | 0.0001055 |
| DDR1 | 2.71758 | 7.7945794 | 1.5201487 | 2.99E-08 | 2.30E-07 |
| CBX2 | 0.5485566 | 1.5755781 | 1.5221688 | 7.66E-11 | 1.75E-09 |
| DRD4 | 0.5575168 | 1.6040181 | 1.5246033 | 0.0062163 | 0.0106699 |
| B3GNT3 | 3.9831632 | 11.48945 | 1.5283233 | 9.72E-08 | 6.29E-07 |
| HK2 | 1.0193449 | 2.9438712 | 1.5300723 | 1.87E-05 | 6.04E-05 |
| TUSC3 | 1.270364 | 3.6718141 | 1.5312511 | 0.0010909 | 0.0022502 |
| MMP7 | 4.0034854 | 11.627705 | 1.5382379 | 0.0005082 | 0.0011418 |
| MACIR | 0.5808237 | 1.6913152 | 1.5419733 | 1.65E-09 | 2.05E-08 |
| KIF2C | 1.610908 | 4.7062441 | 1.5467021 | 5.04E-14 | 6.36E-12 |
| FZD1 | 0.729365 | 2.1379597 | 1.5515217 | 1.77E-09 | 2.18E-08 |
| KLF5 | 1.4862121 | 4.3589564 | 1.5523427 | 1.06E-07 | 6.82E-07 |
| TRIP13 | 0.9192173 | 2.7032538 | 1.5562191 | 3.88E-14 | 5.51E-12 |
| BACE2 | 2.3620278 | 7.0101781 | 1.5694252 | 0.0035455 | 0.006474 |
| SLC1A5 | 4.4404714 | 13.193806 | 1.5710761 | 1.88E-07 | 1.10E-06 |
| TRNP1 | 5.7725604 | 17.189741 | 1.5742646 | 2.98E-07 | 1.62E-06 |
| KIF23 | 0.5973524 | 1.7902509 | 1.5835076 | 9.61E-17 | 1.24E-13 |
| LPAR2 | 0.9380011 | 2.8129137 | 1.5844037 | 6.60E-05 | 0.0001836 |
| BICC1 | 1.9452026 | 5.8577519 | 1.5904267 | 0.000216 | 0.0005358 |
| MISP | 1.6400417 | 4.9548748 | 1.5951161 | 0.0002136 | 0.0005308 |
| DSG2 | 3.481648 | 10.545052 | 1.598724 | 3.20E-14 | 4.71E-12 |
| ANLN | 1.1468063 | 3.4791163 | 1.6010992 | 9.22E-17 | 1.24E-13 |
| SPHK1 | 2.6001866 | 7.9319559 | 1.6090615 | 7.05E-05 | 0.0001949 |
| MELTF | 0.7519323 | 2.2997623 | 1.6128101 | 1.26E-05 | 4.28E-05 |
| MUC5B | 1.8705296 | 5.7261621 | 1.6141217 | 0.0005323 | 0.0011889 |
| PPP1R14D | 0.6067567 | 1.8583868 | 1.6148607 | 4.64E-05 | 0.0001344 |
| PAGE2B | 3.0092296 | 9.2245842 | 1.6160897 | 0.0055225 | 0.0095964 |
| CXCL6 | 2.2025851 | 6.8027852 | 1.6269277 | 0.0008055 | 0.0017178 |
| CEP55 | 0.7213394 | 2.2288384 | 1.6275419 | 1.59E-12 | 8.61E-11 |
| PITX1 | 0.8390025 | 2.6267015 | 1.6465052 | 1.94E-05 | 6.21E-05 |
| ANKRD1 | 1.048535 | 3.2855523 | 1.6477609 | 0.0125565 | 0.0199791 |
| AC245100.3 | 7.2621031 | 22.815676 | 1.6515661 | 0.000489 | 0.0011026 |
| AFAP1-AS1 | 0.6431888 | 2.0239087 | 1.65383 | 0.0006831 | 0.0014827 |
| SLC16A3 | 1.4773241 | 4.6732578 | 1.6614422 | 6.11E-08 | 4.25E-07 |
| TGFA | 0.6649214 | 2.103789 | 1.6617343 | 0.000172 | 0.0004357 |
| AC016735.1 | 1.0580993 | 3.3964715 | 1.6825617 | 3.32E-05 | 0.0001001 |
| C1orf116 | 0.5815096 | 1.8674492 | 1.6831941 | 1.70E-08 | 1.43E-07 |
| G6PD | 6.4073275 | 20.626836 | 1.6867279 | 1.31E-09 | 1.69E-08 |
| IGSF3 | 1.1264947 | 3.6277918 | 1.6872511 | 8.10E-13 | 5.07E-11 |
| PLPP2 | 1.8339612 | 5.9138117 | 1.6891252 | 1.58E-05 | 5.20E-05 |
| C6orf223 | 0.8163624 | 2.6400884 | 1.6933046 | 1.82E-09 | 2.22E-08 |
| CDCA7 | 0.6185063 | 2.0029067 | 1.695235 | 2.92E-05 | 8.92E-05 |
| SPINT1 | 5.2768487 | 17.089814 | 1.6953882 | 6.39E-05 | 0.000178 |
| PTGFR | 0.829371 | 2.7162089 | 1.711505 | 8.63E-05 | 0.0002344 |
| DEPDC1B | 0.7949376 | 2.611208 | 1.7158038 | 9.12E-14 | 9.91E-12 |
| DNER | 0.5440134 | 1.794315 | 1.721719 | 2.84E-05 | 8.73E-05 |
| MYBL2 | 4.2992484 | 14.275145 | 1.731349 | 7.98E-13 | 5.07E-11 |
| SEMA6A | 0.8187025 | 2.740933 | 1.7432559 | 7.02E-05 | 0.0001942 |
| NEURL3 | 1.2908227 | 4.3407432 | 1.7496512 | 3.59E-06 | 1.42E-05 |
| ERP27 | 0.5012524 | 1.6930944 | 1.7560534 | 0.0013657 | 0.0027612 |
| GAL3ST1 | 3.0288081 | 10.275682 | 1.7624121 | 2.55E-08 | 2.01E-07 |
| NPTX2 | 2.3330656 | 7.9285425 | 1.7648288 | 0.0017655 | 0.0034685 |
| MATN3 | 0.631462 | 2.1550808 | 1.7709742 | 0.0008093 | 0.0017241 |
| NCEH1 | 1.2798902 | 4.3731517 | 1.7726534 | 5.34E-08 | 3.77E-07 |
| EPHB6 | 0.6914885 | 2.3763173 | 1.7809504 | 4.92E-05 | 0.0001413 |
| SNAP25 | 0.8282141 | 2.8532325 | 1.7845217 | 0.0018436 | 0.0036033 |
| TMC5 | 0.8368467 | 2.8884527 | 1.7872616 | 0.0044204 | 0.007874 |
| HSPA7 | 0.9710442 | 3.3566499 | 1.7894132 | 0.0007438 | 0.001599 |
| KRT17 | 0.9073745 | 3.1437095 | 1.7926979 | 0.0057038 | 0.0098849 |
| CLDN4 | 4.1851346 | 14.514525 | 1.7941514 | 6.62E-09 | 6.51E-08 |
| MAEL | 0.500443 | 1.7644908 | 1.8179742 | 3.57E-08 | 2.67E-07 |
| B3GNT7 | 0.5553702 | 1.9609169 | 1.8200066 | 0.000688 | 0.0014919 |
| SCRN1 | 0.9919017 | 3.5039468 | 1.8207117 | 2.57E-05 | 7.97E-05 |
| RHEX | 0.5818506 | 2.0589173 | 1.8231653 | 0.0003272 | 0.0007722 |
| PFKP | 2.1507413 | 7.624088 | 1.8257308 | 0.0010723 | 0.0022154 |
| PKM | 11.121455 | 39.564569 | 1.8308635 | 5.09E-11 | 1.26E-09 |
| WNK2 | 0.5934024 | 2.1636499 | 1.8663844 | 6.71E-06 | 2.46E-05 |
| HSPA6 | 1.8551308 | 6.7934432 | 1.8726221 | 4.36E-05 | 0.0001273 |
| CACNG4 | 0.7871992 | 2.8831606 | 1.8728505 | 2.79E-05 | 8.57E-05 |
| CRYGS | 0.6757577 | 2.4787733 | 1.8750485 | 7.40E-05 | 0.0002036 |
| ITGB4 | 1.5146053 | 5.661024 | 1.9021212 | 5.07E-06 | 1.92E-05 |
| CTSV | 0.5859079 | 2.234717 | 1.9313462 | 6.20E-10 | 9.10E-09 |
| SLC7A10 | 0.4163098 | 1.6113986 | 1.952584 | 0.0132319 | 0.0209317 |
| MAB21L2 | 0.4523159 | 1.7510321 | 1.9528029 | 0.0191714 | 0.0292238 |
| ITGA3 | 1.087797 | 4.4364592 | 2.0279994 | 0.0086815 | 0.0143431 |
| EGLN3 | 0.6211177 | 2.5451993 | 2.0348399 | 4.73E-12 | 1.97E-10 |
| COL9A2 | 0.4961023 | 2.0409023 | 2.0404975 | 5.27E-09 | 5.46E-08 |
| NXPH4 | 1.2644752 | 5.2601198 | 2.0565569 | 1.27E-06 | 5.69E-06 |
| ITPR3 | 0.6130404 | 2.5658164 | 2.065364 | 9.32E-08 | 6.07E-07 |
| CTNND2 | 0.6778147 | 2.8574614 | 2.0757712 | 2.37E-06 | 9.80E-06 |
| CRABP2 | 0.6260394 | 2.6975203 | 2.1073085 | 0.0002279 | 0.0005618 |
| ELOVL7 | 0.9132303 | 3.9911659 | 2.1277596 | 6.79E-10 | 9.73E-09 |
| PAGE2 | 3.0854283 | 13.570263 | 2.136906 | 0.0014627 | 0.002934 |
| POF1B | 0.4225894 | 1.8601001 | 2.1380519 | 2.57E-06 | 1.05E-05 |
| TNFRSF11B | 1.174257 | 5.223775 | 2.1533446 | 5.47E-17 | 1.24E-13 |
| PRAME | 1.1127764 | 4.9776341 | 2.1612965 | 2.48E-05 | 7.74E-05 |
| CTSE | 0.6739743 | 3.0322646 | 2.1696301 | 4.47E-05 | 0.0001301 |
| B4GALNT4 | 0.3968501 | 1.7855656 | 2.1697152 | 0.0248752 | 0.037003 |
| PLEKHB1 | 0.6057928 | 2.7571545 | 2.1862838 | 9.36E-05 | 0.0002514 |
| EPO | 1.036834 | 4.7559039 | 2.1975347 | 6.31E-05 | 0.0001761 |
| S100A9 | 17.040988 | 78.291715 | 2.1998506 | 0.0114998 | 0.0184599 |
| LINC02041 | 0.3913981 | 1.8095935 | 2.2089569 | 2.53E-11 | 7.16E-10 |
| TMEM132A | 0.9304622 | 4.3232009 | 2.2160805 | 0.0001947 | 0.0004876 |
| LTO1 | 1.007487 | 4.6813613 | 2.2161668 | 4.10E-08 | 3.01E-07 |
| CA9 | 4.1447457 | 19.66795 | 2.2464911 | 1.19E-12 | 6.77E-11 |
| KRT80 | 0.4915303 | 2.3429368 | 2.2529657 | 1.09E-05 | 3.76E-05 |
| FCGBP | 0.4036583 | 1.9391035 | 2.2641834 | 7.84E-08 | 5.26E-07 |
| TUBA3C | 0.9223329 | 4.5196132 | 2.2928398 | 0.0002811 | 0.0006745 |
| FLNC | 0.9451399 | 4.6359875 | 2.2942769 | 4.66E-06 | 1.78E-05 |
| AC105118.1 | 0.5011712 | 2.5162085 | 2.3278762 | 0.0009388 | 0.0019706 |
| C12orf75 | 1.9832666 | 9.9807578 | 2.3312707 | 4.75E-14 | 6.15E-12 |
| FOLR1 | 0.3681568 | 1.8698126 | 2.3445012 | 8.56E-09 | 8.13E-08 |
| AGR2 | 3.7819785 | 20.730691 | 2.4545551 | 1.04E-06 | 4.78E-06 |
| LINC01436 | 0.5110935 | 2.8834846 | 2.496154 | 0.0053823 | 0.009392 |
| TFF1 | 1.7216832 | 9.8303328 | 2.5134205 | 4.24E-06 | 1.64E-05 |
| FXYD3 | 0.86393 | 4.9495169 | 2.5183014 | 5.51E-07 | 2.76E-06 |
| ARL14 | 0.5325634 | 3.1771727 | 2.5767183 | 0.0033284 | 0.0061005 |
| GCNT3 | 0.406891 | 2.4763659 | 2.6055102 | 4.46E-09 | 4.73E-08 |
| ACTL8 | 0.3386415 | 2.1501923 | 2.6666348 | 0.0050313 | 0.0088418 |
| KRT19 | 3.8714816 | 24.864519 | 2.6831308 | 0.0058803 | 0.0101561 |
| CHST4 | 0.4219992 | 2.7112059 | 2.6836224 | 0.0020258 | 0.0039145 |
| CAPN6 | 0.4138115 | 2.696516 | 2.7040509 | 9.54E-05 | 0.0002559 |
| SGPP2 | 0.3161236 | 2.0932708 | 2.7271984 | 4.91E-06 | 1.87E-05 |
| LAMC2 | 0.414567 | 2.7941475 | 2.7527311 | 0.0010253 | 0.002129 |
| PTHLH | 0.2994201 | 2.1623317 | 2.8523447 | 1.48E-06 | 6.51E-06 |
| DUOXA2 | 0.6120817 | 4.7631534 | 2.9601208 | 0.0091446 | 0.0150272 |
| AC010547.2 | 0.254966 | 2.0229574 | 2.988089 | 0.000868 | 0.0018373 |
| SYT13 | 0.4219384 | 3.3618204 | 2.9941384 | 0.0002326 | 0.000572 |
| FOXJ1 | 0.3107513 | 2.521884 | 3.0206697 | 2.31E-12 | 1.12E-10 |
| SFRP5 | 1.604842 | 13.412768 | 3.0631038 | 0.0177662 | 0.0273215 |
| MMP1 | 0.4160732 | 3.5704422 | 3.1011934 | 3.79E-07 | 1.99E-06 |
| RHOV | 0.2777995 | 2.5051114 | 3.1727587 | 1.19E-05 | 4.08E-05 |
| CXCL5 | 0.6710076 | 6.1971155 | 3.2071959 | 1.11E-05 | 3.84E-05 |
| SMIM22 | 0.254932 | 2.371955 | 3.2178923 | 5.55E-09 | 5.70E-08 |
| PKP3 | 0.238557 | 2.2624318 | 3.2454681 | 1.22E-05 | 4.17E-05 |
| LINC00942 | 0.29024 | 2.8406153 | 3.2908852 | 0.0006712 | 0.0014602 |
| CLDN10 | 0.3422295 | 3.6854717 | 3.4288132 | 0.0009668 | 0.0020212 |
| PRSS22 | 0.1719744 | 1.8732175 | 3.445253 | 0.0010304 | 0.0021383 |
| MUC1 | 0.3443463 | 3.800684 | 3.4643269 | 8.39E-05 | 0.0002287 |
| SCTR | 0.4531528 | 5.3567176 | 3.5632798 | 0.0002135 | 0.0005307 |
| PROM1 | 0.1572528 | 2.0069709 | 3.6738619 | 0.0004176 | 0.0009592 |
| CFTR | 0.2911696 | 3.7789472 | 3.6980525 | 6.36E-05 | 0.0001774 |
| FGF19 | 1.0219056 | 14.070661 | 3.7833563 | 0.0232767 | 0.0348925 |
| DUOX2 | 0.5786426 | 8.4496018 | 3.8681388 | 0.0047955 | 0.0084759 |
| SYT8 | 0.1696139 | 2.9048627 | 4.0981435 | 1.89E-07 | 1.11E-06 |
| VTCN1 | 0.2752624 | 5.130728 | 4.2202842 | 4.15E-05 | 0.0001218 |
| TFF2 | 0.4504931 | 8.8043305 | 4.2886365 | 2.19E-06 | 9.16E-06 |
| MSLN | 0.0932102 | 1.9878453 | 4.4145734 | 0.0107099 | 0.0173473 |
| SLC34A2 | 0.423335 | 10.496982 | 4.6320309 | 0.001482 | 0.0029686 |
| UCA1 | 0.17385 | 4.4852083 | 4.68926 | 0.0002581 | 0.0006268 |
| CHGA | 0.0453316 | 4.2282009 | 6.5433833 | 0.0023824 | 0.0045233 |
| PRSS2 | 0.1084791 | 15.710791 | 7.1781951 | 0.0024132 | 0.0045735 |
| CPA2 | 0.0258683 | 7.1520557 | 8.1110314 | 0.0004548 | 0.0010335 |
