# Supplementary figures and images for: Identification and validation a costimulatory molecule gene signature to predict the prognosis and immunotherapy response for hepatocellular carcinoma
Source: Cancer Cell Int. 2022 Feb 22;22:97. doi: 10.1186/s12935-022-02514-0 (PMC8864933; doi:10.1186/s12935-022-02514-0)

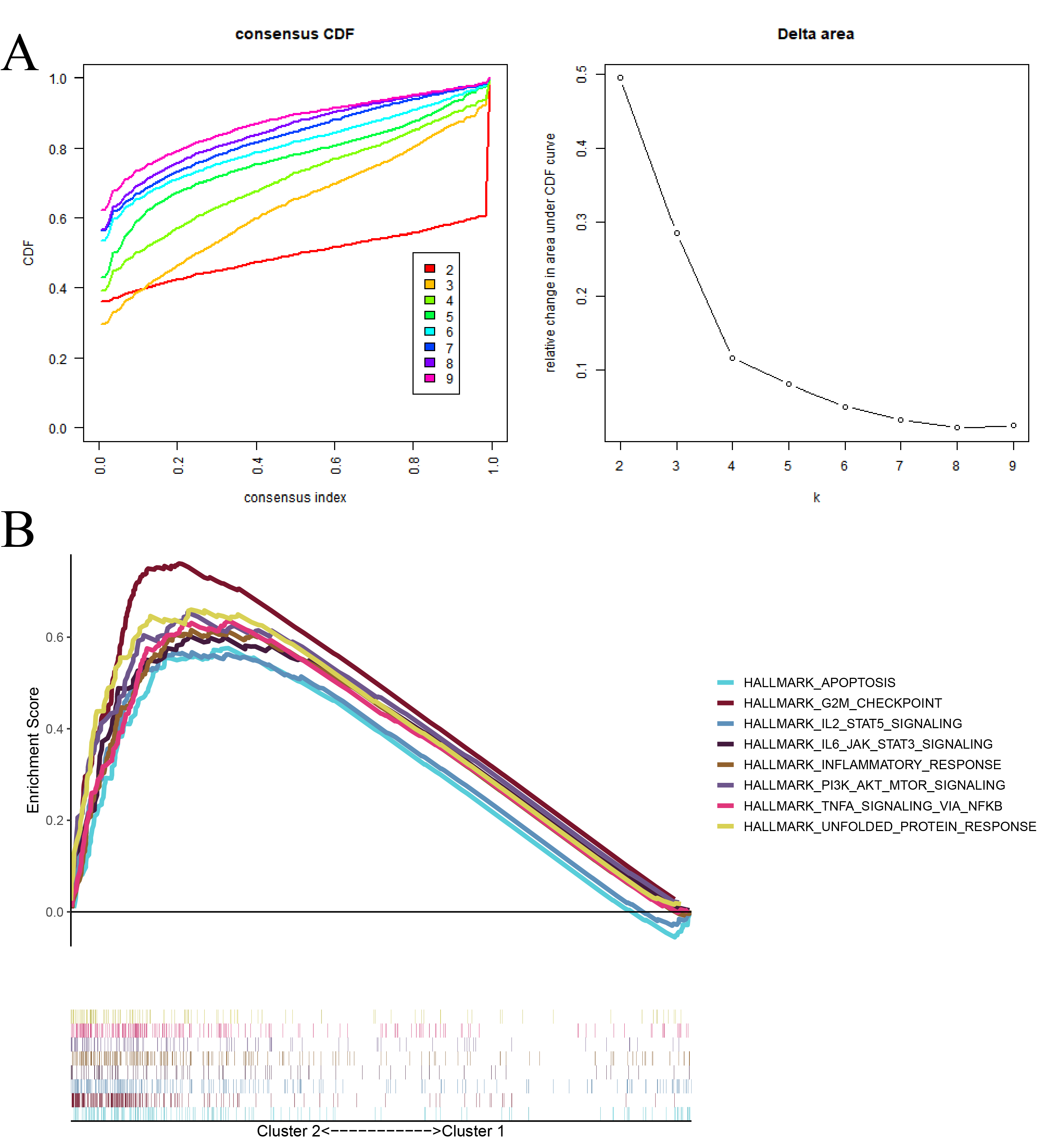

Supplement: Supplementary file 3 — Additional file 3: Figure S1: Consensus clustering based on the CMGs. (A) Cumulative distribution function (CDF) curves in consensus clustering and relative changes in area under CDF curves for k = 2 to 10. (B) The Gene Set Enrichment Analysis of the oncogenic pathways in Cluster 2. [file 12935_2022_2514_MOESM3_ESM.tif]

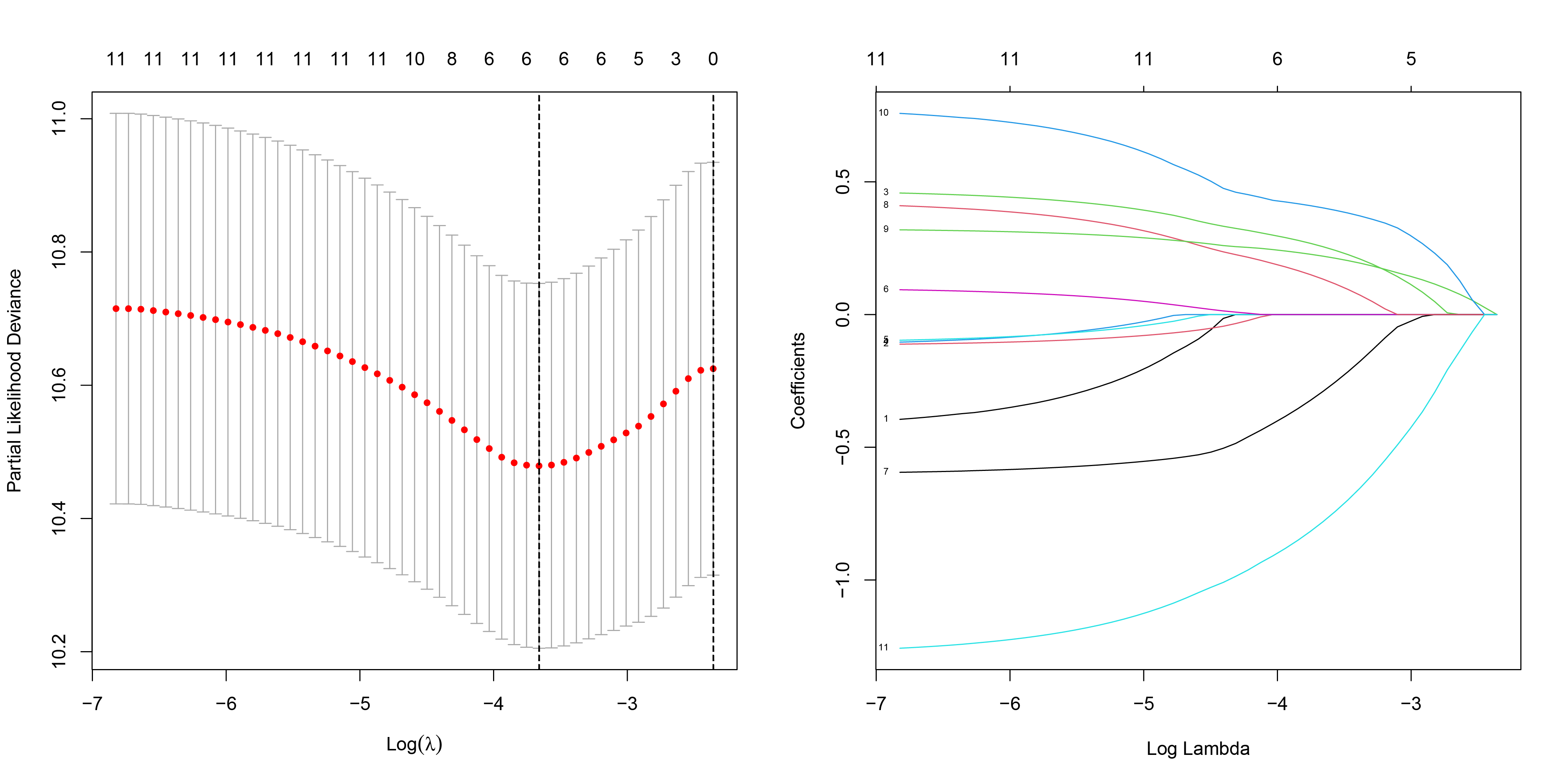

Supplement: Supplementary file 4 — Additional file 4: Figure S2: A LASSO Cox regression analysis further fine-turned the selection of CMGs-related risk signature. [file 12935_2022_2514_MOESM4_ESM.tif]

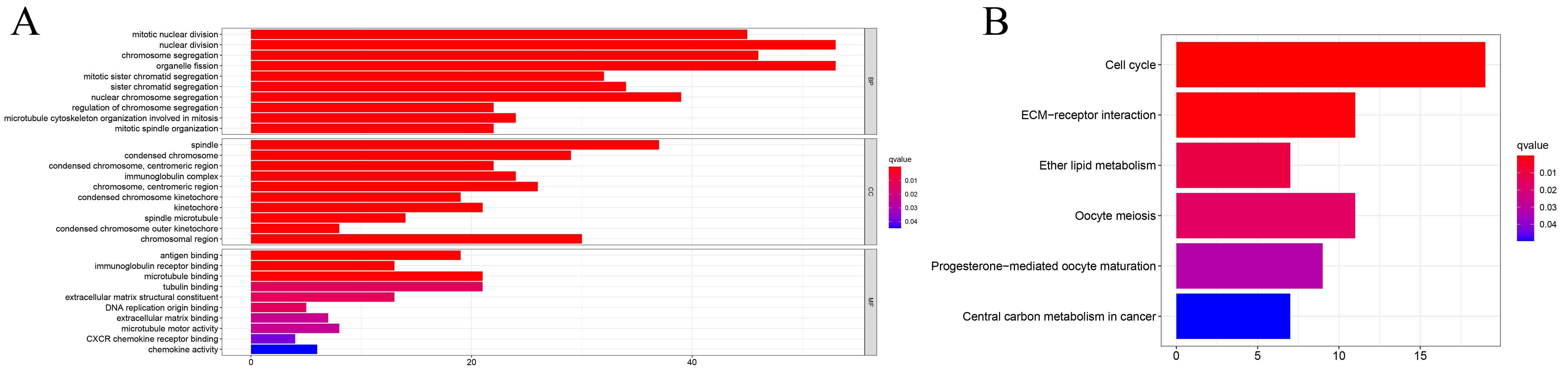

Supplement: Supplementary file 5 — Additional file 5: Figure S3: Functional enrichment analysis of the DEGs. (A) Gene ontology analysis. (B) Kyoto Encyclopedia of Genes and Genomes pathways. [file 12935_2022_2514_MOESM5_ESM.tif]

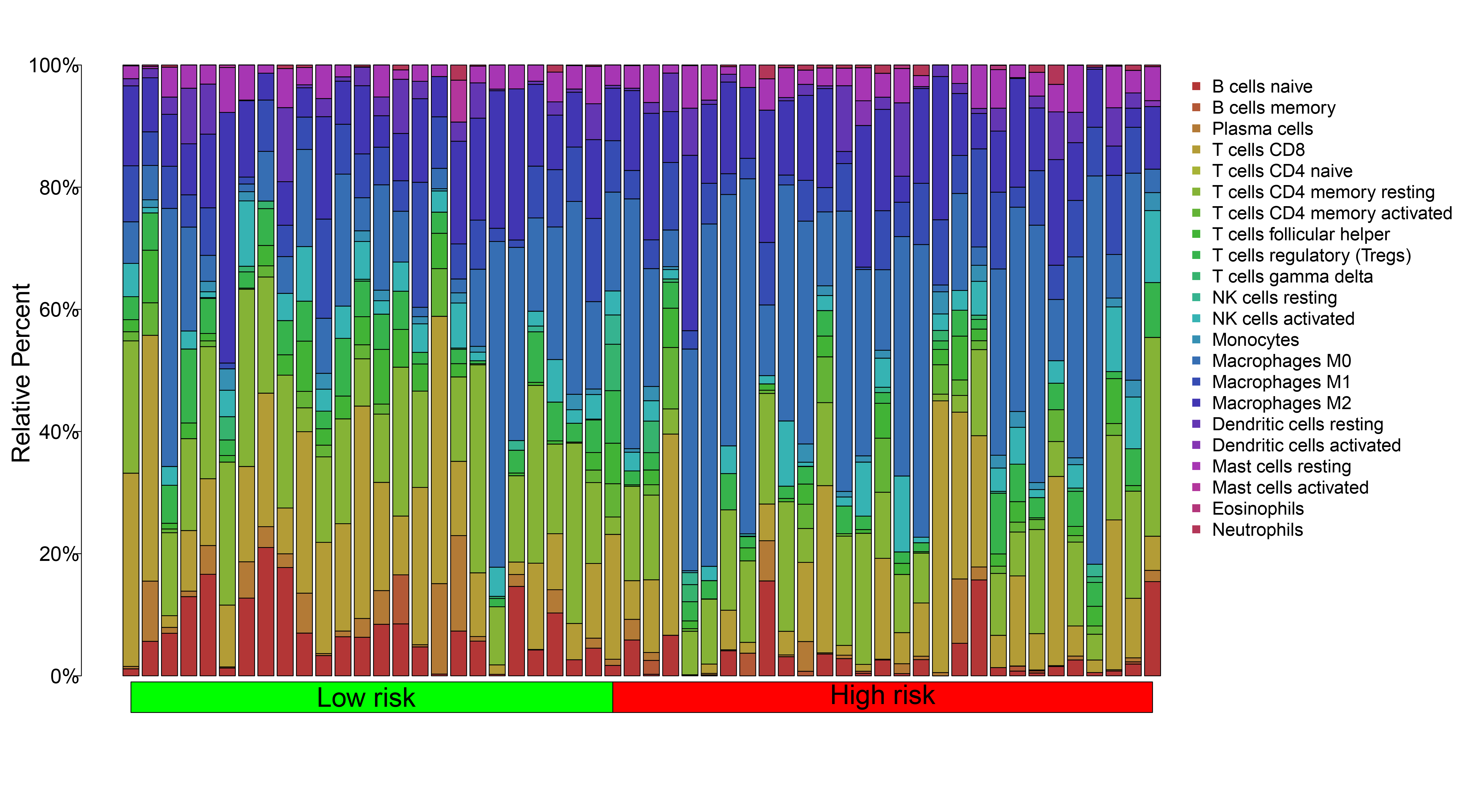

Supplement: Supplementary file 6 — Additional file 6: Figure S4: The abundance of 22 immune cells between the high- and low-risk groups. [file 12935_2022_2514_MOESM6_ESM.tif]
